# Lung cancer in idiopathic pulmonary fibrosis: A systematic review and meta-analysis

**DOI:** 10.1371/journal.pone.0202360

**Published:** 2018-08-16

**Authors:** AliReza JafariNezhad, Mohammad Hossein YektaKooshali

**Affiliations:** 1 Inflammatory Lung Disease Research Center, Department of Internal Medicine, Razi Hospital, School of Medicine, Guilan University of Medical Sciences, Rasht, Iran; 2 Student Research Committee, School of nursing, Midwifery and Paramedicine, Guilan University of Medical Sciences, Rasht, Iran; Istituto di Ricovero e Cura a Carattere Scientifico Centro di Riferimento Oncologico della Basilicata, ITALY

## Abstract

**Background:**

There are many epidemiological pieces of evidence that show IPF patients have the highest risk of lung cancer. We conducted a systematic review of all published data to define the characteristics of lung cancer that develops in IPF by performing a meta-analysis.

**Method:**

This study was performed based on the PRISMA guideline. Documents gathered by searching through the Web of Sciences, Scopus, PubMed/Medline, OVID, and COCHRANE databases which published before 03/25/2018 that related to lung cancer in IPFs’ patients. Articles were searched using standard keywords as well as Mesh and Mesh Entry and all probabilistic combinations of words using Boolean operators. Data searching, extracting and quality appraising were done by two researchers, independently. At last, Random-effects size based on Cochrane test and I^2^ were used. The review protocol has been registered in PROSPERO with ID: CRD42018094037.

**Results:**

Based on the meta-analysis conducted in 35 (0.18%) included studies, the total sample size of patients with IPF was estimated 131947 among whom 6384 had LC. The total rate of LC prevalence in IPF patients was estimated to be 13.54% (95% CI: 10.43–17.4) that was significantly 9 times higher in men vs. Women and smoker vs. non-smoker. Highest to lowest prevalence of cellular (histological) subtypes of lung cancer in IPF were SQCC (37.82%), ADC (30.79%), SmCC (20.48%), LCC (5.21%), and ADQC (4.81%), respectively. The highest and lowest stage of lung cancer in IPF patients was estimated at III and II, respectively. The highest involvement location of lung cancer in IPF patients was in the Peripheral. Also, the prevalence of the tumor region involved from the highest to the lowest was estimated to be in the RLL, LLL, RUL and LUL regions.

**Conclusions:**

Lung cancer in IPF, most commonly SQCC, presents in elderly heavy smokers with a male, locating in peripheral regions and the lower part of lung predominance.

## 1. Introduction

Idiopathic Pulmonary Fibrosis (IPF), known as Cryptogenic Fibrosing Alveolitis (CFA), diffuse Fibrosing Alveolitis (diff FA), and Usual Interstitial Pneumonitis (UIP) is one of the most common forms of Interstitial Lung Disease (ILD) over the years [1–6]. IPF is an unknown chronic pulmonary disease with unknown origin. It is a chronic lung disease characterized by a progressive and irreversible decline in lung function [1, 7]. Due to the increasing prevalence of this disease in the United States of America, 48,000 new cases are diagnosed each year, with an annual mortality rate of 40,000 individuals (84%), which is as many as the mortality rate of breast cancer [8]. The incidence of IPF rises exponentially in the 50–85-year-old age groups (only 2–15% prevalence in patients under 50 [2, 9, 10]) [11–15].

Family history of disease (contrary to the common form, the prevalence in younger ages is higher[16–18])[16, 17, 19, 20], male gender, smoking, various types of environmental effects such as organic and inorganic dust, medical treatments, other medical disorders and microbial agents like Epstein-Barr viruses are risk factors that increase the risk of IPF [21]. Age over 50 years, dry and non-productive cough on exertion, progressive exertional dyspnea, dry inspiratory bibasilar crackles “Velcro-like” on auscultation, clubbing of the digits, hypoxemia, and abnormal results of lung function test and, constraint evidence and disturbance in gas exchange are symptoms, clinical and diagnostic features of IPF [11, 14, 15, 22].

Among radiographic features used in screening IPF patients, chest x-ray (CXR) can be mentioned, which the diagnosis and clinical manifestations may show bilateral interstitial opacities with the possibility of occurrence in peripheral and lower lung zones. This demonstration may not be visible in 2 to 10% of patients [1, 11, 22]. Also, HRCT is very important and crucial due to the high sensitivity and specificity which can differentiate IPF from other ILDs [1, 23, 24]. According to the ATS / ERS / JRS / ALAT 2011 protocol, the HRCT is the essential test for diagnosing IPF. Reticular opacities (mostly associated with bronchiectasis), honeycombing manifested (typically with 3–10 mm, occasionally large, and usually sub-pleural with well-defined walls), ground-glass opacities (more common, but less than reticulation) and distribution, particularly is in the basal and peripheral and often scattered areas that can be the diagnosis and clinical manifestations in the HRCT test of patients with IPF[1]. Also, forced vital capacity (FVC) ≥50%, diffusing capacity for carbon monoxide (DLCO) ≥ 30% and 6-minute walk test (6MWT) distance≥ 150 meters in the spirometry test represent the mild-to-moderate level of IPF disease [25–28]. Likewise, in laboratory testing issued for screening patients with ILDs, including IPF, the Krebs von den Lungen 6 (KL-6) biomarker is referred, which is not widely used[29, 30]. Since the diagnosis of IPF involves clinical, radiological and histopathological findings, the multi-diagnostic test increases the accuracy[31].

Among, lung cancer(LC)[32], pulmonary hypertension (PH)[33], obstructive sleep apnea syndrome[34], gastroesophageal reflux[35], coronary heart disease[1], pulmonic component of the second heart sound, right ventricular lift and tricuspid regurgitation[36, 37] can be mentioned as the comorbidities and common complications in patients with IPF. And, despite numerous conducted studies their correlation and cause have not been explicitly and directly specified[38].

There are many epidemiological pieces of evidence that show IPF patients have the highest risk of lung cancer[39, 40], that has been reported more often in older men smokers[8, 41–44]. Lung cancer detection in IPF patients due to fibrotic changes in the lung is difficult, which appears more often as nodular lesions with irregular or spiculated margins in peripheral lung zones[45, 46] that its diagnosis can be evaluated before, during and after treatment[8, 41–44]. The risk of IPF patients with LC during surgical treatment is much higher than those without IPF[47] that in severe cases causes acute respiratory distress syndrome[48, 49].

Despite previously published studies about the prevalence of LC in IPF patients in different countries (primary study) [3–6, 41, 44, 50–79], no comprehensive study and systematic review and meta-analysis have been conducted globally. One of the most important goals of systematic review and meta-analysis studies is combining the existing studies to increase sample size due to the increased number of related studies and to reduce the differences between the existing parameters and confidence interval, which ultimately leads to solving the review problems in the previous method. Certainly, such studies are a vital link between research studies and clinical decision making at the patient's bedside[80–86]. Considering the above mentioned cases and the prevalence, severity and extent of LC in IPF patients, as well as the presentation of the final conclusions for policy-making and correct management planning at the macro level, a systematic review of all documentation and their combination, is conducted via meta-analysis method to estimate the overall rate of LC in IPF patients and other risk factors.

## 2. Materials and methods

### 2.1. Study protocol

The present study is based on the Meta-analysis of Observational Studies in Epidemiology guideline [87] and it has been conducted in 5 steps according to the PRISMA statement[88] (S1 File) including design and search strategy, a collection of articles and their systematic review, evaluation of inclusion and exclusion criteria, qualitative evaluation and statistical analysis of data. All steps were carried out by two researchers independently and, any encounters were assessed by a specialist. The review protocol has been registered in PROSPERO: International Prospective Register of Systematic Reviews (https://www.crd.york.ac.uk/PROSPERO/) Identifier: CRD42018094037 [89, 90](S2 File).

### 2.2. Search strategy

An advanced relevant search was conducted in international databases, such as Web of Sciences, Scopus, PubMed/Medline, OVID, and COCHRANE, to collect all of the studies which were related to LC in IPF patients. Articles were searched using standard keywords as well as Mesh and Mesh Entry and all probabilistic combinations of words using Boolean operators combined in accordance with the search syntax (S1 Table) on each database without time limit until 03/25/2018. And, a manual search was also done as reviewing the reference list of related articles. The important point in searching the databases was the high-sensitivity searching, and also the search was conducted by the researchers and a specialist which is expert in searching databases (A.R).

### 2.3. Inclusion and exclusion criteria

#### 2.3.1. Inclusion criteria based on PICO (related to Evidence-Based Medicine) [91]

(1) **P**opulation: Cohort and retrospective studies that investigated LC in IPF patients; (2) **I**ntervention: Surgical resection or radiological and pathologically confirmed cancer; (3) **C**omparison: That can show the rate of LC incidence in relation to non-LC in patients with IPF, which is called prevalence rate; (4) **O**utcome: Estimate the overall rate of LC in IPF patients and other risk factors.

#### 2.3.2. Exclusion criteria

(1) Review articles, Letters, Comments, Case reports (only for estimating overall prevalence), or Conference proceedings; (2) Studies that did not focus on the prevalence of LC in IPF patients; (3) Duplicated papers; (4) Non-English full text; (5) Non-accessible full text.

### 2.4. IPF and LC detection criteria

#### 2.4.1. IPF detection criteria

All patients after initial diagnosis of IPF based on CT-Scan and pathological criteria by examining the dossier or attending the clinic, according to the American Thoracic Society (ATS) and the European Respiratory Society (ERS)[1, 92, 93] and the official ATS / ERS / Japanese Respiratory Society / Latin American Thoracic Society statement on IPF[13, 94, 95] were also tested for their IPF disease (and other parameters of Pulmonary function tests such as: forced vital capacity (FVC) of ≥50% and DLCO of ≥30% and 6-minute walk test (6MWT) distance ≥150 meters[25–28]).

#### 2.4.2. LC detection criteria

To diagnose the LC in patients, after presuming IPF, the pathology and cytology reports were evaluated and categorized into two groups of patients with and without LC. Cellular (histological) subtypes and clinical staging and region and location of LC were also evaluated in patients[3, 6, 55, 96].

### 2.5. Selection of studies

**Fig 1:**
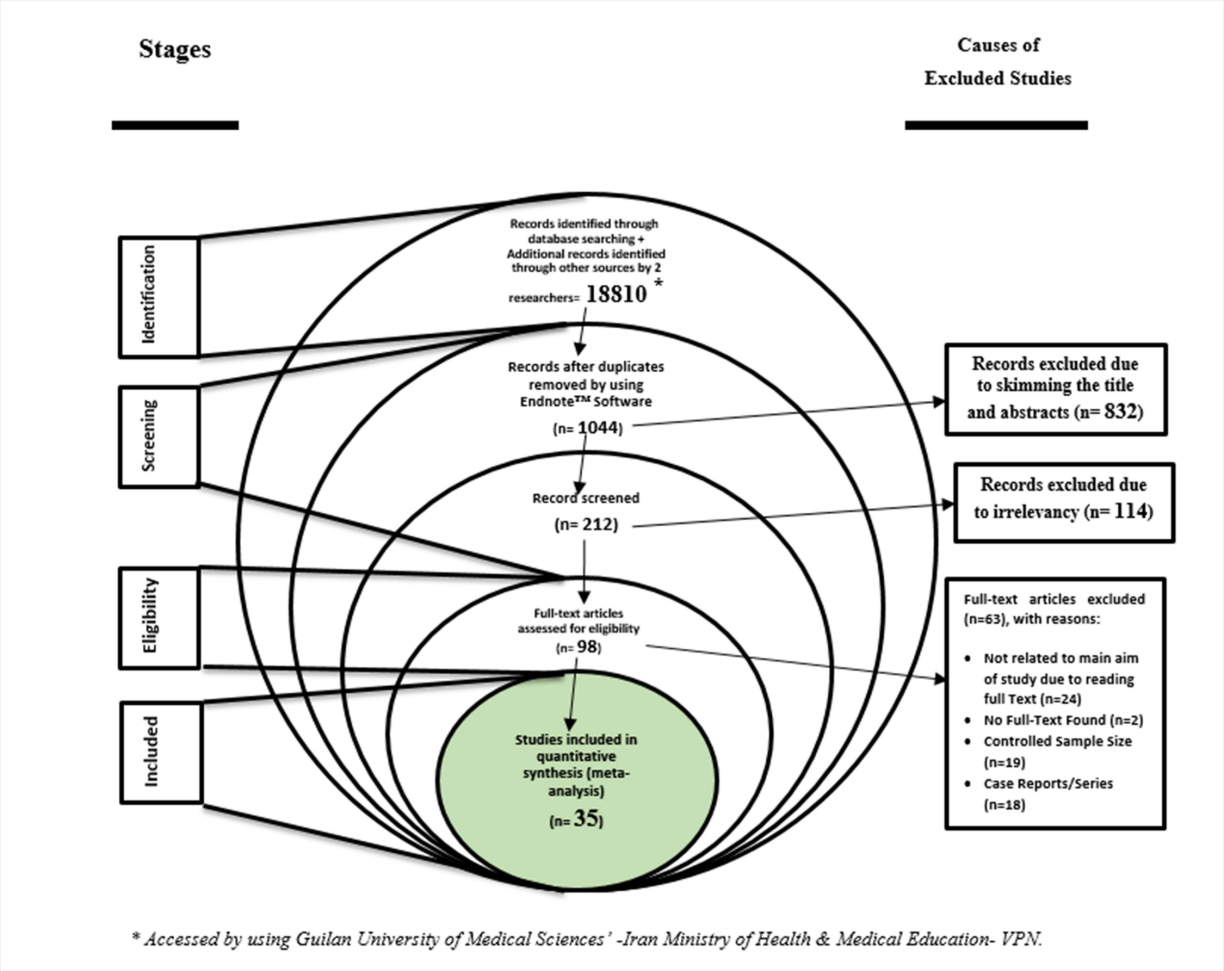
A flow diagram (Stacked Venn) following the “PRISMA flow chart”.

After the end of the search, the papers were entered into the EndNote, reference management software and, after “Find References Updates”, duplicates were removed. After blinding studies (hiding authors', the name of the journal and published year), each study was evaluated by two researchers independently in the screening stage, with skimming and scanning the study titles, to evaluate the inclusion and exclusion criteria and IPF and LC detection criteria (the eligibility stage). In the event of disagreement between the two researchers, the specialist researcher made the final decision (Fig 1).

### 2.6. Quality appraisal

After excluding irrelevant studies in the screening and eligibility stages, the quality of the final studies was examined. At this stage, the Newcastle-Ottawa Scale (NOS)[97] checklist (S3 File) was used which consists of 8 sections, and divides the studies with a scale score of 0 to 8 from poor to high quality, respectively. According to this scoring, the studies are divided into three levels of scoring: 1- Studies with a score of 5: poor quality; 2- studies with a score of 5–6: medium quality; 3- studies with a score of 7 to 8: high quality. Finally, (at the included stage), the articles that had medium to high quality were entered into the next stage (Fig 1).

### 2.7. Data extraction

At first, a checklist was designed according to the aims of the study and studying other available resources. Designed checklist includes items: Author name, Year, Place, Sample Size(SS), Periods of time, SS of IPF, SS of LC, Prevalence (LC in IPF), Cellular (Histological) Subtypes (ADC(Adenocarcinoma (%)), SQCC(Squamous-cell carcinoma (%)), SmCC(Small-cell carcinoma (%)), LCC(large-cell carcinoma (%)), ADSQC(Adeno-Squamous carcinoma (%)), Others), Clinical Staging (I, II, II, IV), Prevalence separated by Sex, Prevalence separated by Smoking status, FEV1(Forced Expiratory Volume in the first second) (Mean±SD), FVC1(Forced Vital Capacity in the first second) (Mean±SD), FEV1/FVC1 (Mean±SD), Age (Mean±SD), DLco(Diffusing capacity for carbon monoxide) (Mean±SD), Region (RUL(Right Upper Lobe), LUL(Left Upper Lobe), RLL(Right Lower Lobe), LLL(Left Lower Lobe), Upper, Center, Lower) and Location (Peripheral and Central), which were extracted by two independent researchers and blind for the name of the author, institute and journal. In necessary cases, further information and raw data were requested by contacting the author (the first author or responsible or the authors' department).

### 2.8. Statistical analysis

In each study, after considering the prevalence rate of LC in IPF patients as a binomial distribution probability, its variance was calculated by binomial distribution and for evaluating the heterogeneity of the studies Cochran test (Q) and I^2^ index were used. The I^2^ index less than 25% is low heterogeneity, between 75% -25% is the average heterogeneity and more than 75% are considered as heterogeneous[85, 98]. According to the heterogeneity of studies (high), the random effects model has been used to combine the results of studies. Sensitivity analysis (“One Study Removed” test) was conducted to investigate the impact of each study on total results for the overall prevalence and each of the risk factors. In order to evaluate the cause of heterogeneity, the subgroup analysis was performed based on the country, and gender. The Meta-Regression model was used to determine the prevalence rate based on the year of publication. The Egger and Begg's test were evaluated to examine the publication bias (by using the Funnel Plot). Data analysis was performed using the Comprehensive Meta-Analysis Ver.2, and the significance level of the test was considered less than 0.05.

## 3. Results

### 3.1. Search results and characteristics

In this systematic study, based on performed searches, 667 articles were identified and after conclusive investigation and evaluation according to the checklist, 35 (0.18%) articles[3–6, 44, 50–79](S2 Table)(Fig 1) were entered into the final list. The total sample size was estimated to be 38184 patients, with 131947 IPF patients, of which 6384 had LC. The mean age of patients, DLco, FEV, FVC and FEV1/ FVC1 was estimated to be 69.06±2.57, 59.61±12.52, 83.82±6.36, 85.44±6.44 and 76.9±6.47, respectively.

### 3.2. Overall prevalence of LC in IPF

**Fig 2:**
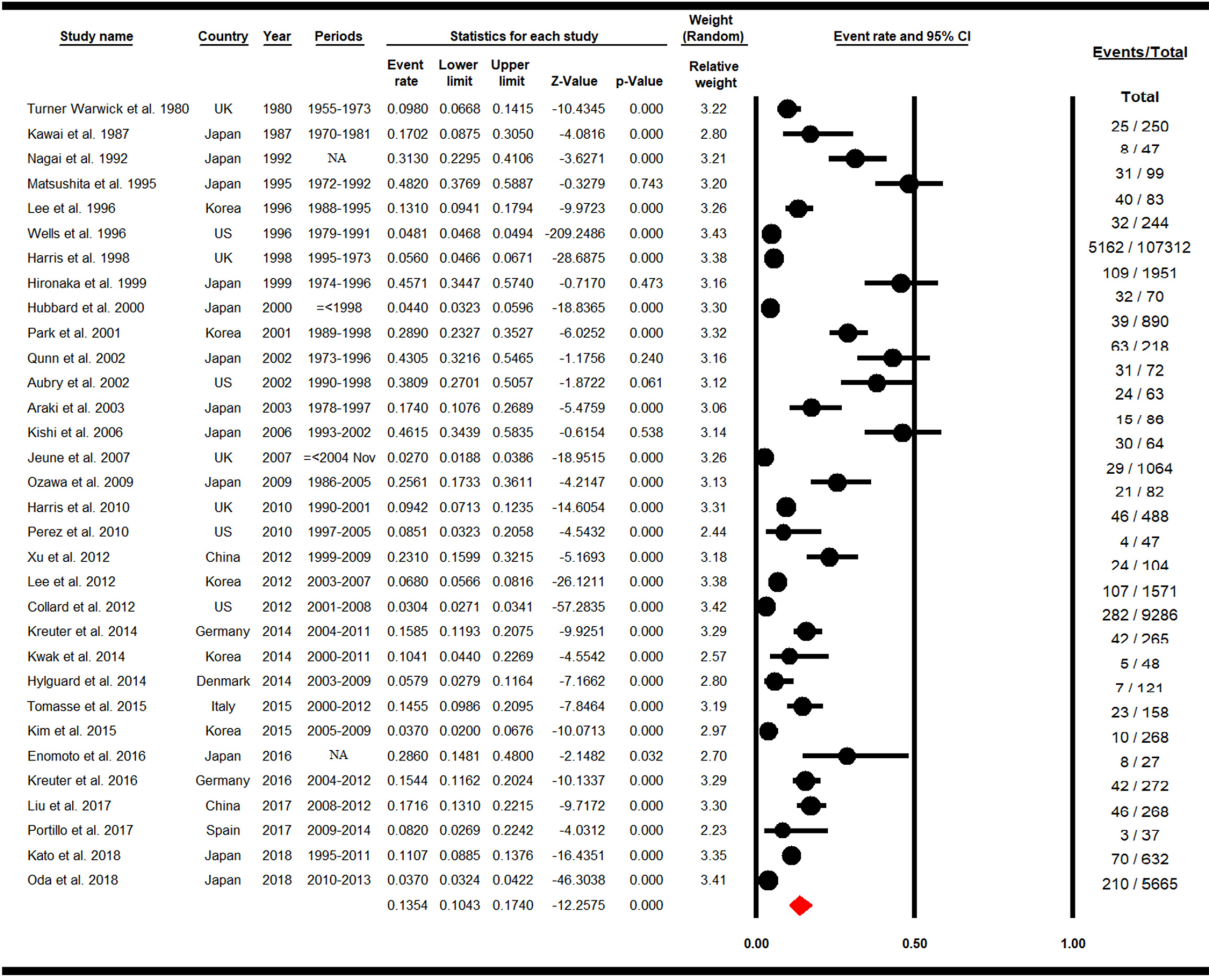
Forest plot for overall prevalence of LC in IPF. (Random effects model).

The overall prevalence rate of LC in patients with IPF was calculated at 13.54% (95% Confidence Interval (CI): 10.43–17.4, P = 0.000). The highest and lowest prevalence rate was observed in Matsushita et al. (1995) from Japan with a prevalence rate of 48.2% (95% CI: 37.69–58.87) and Jeune et al. (2007) from the UK with a prevalence rate of 2.7% (95% CI: 1.88–3.86), respectively (Fig 2).

### 3.3. Prevalence of LC in IPF based on country

**Fig 3:**
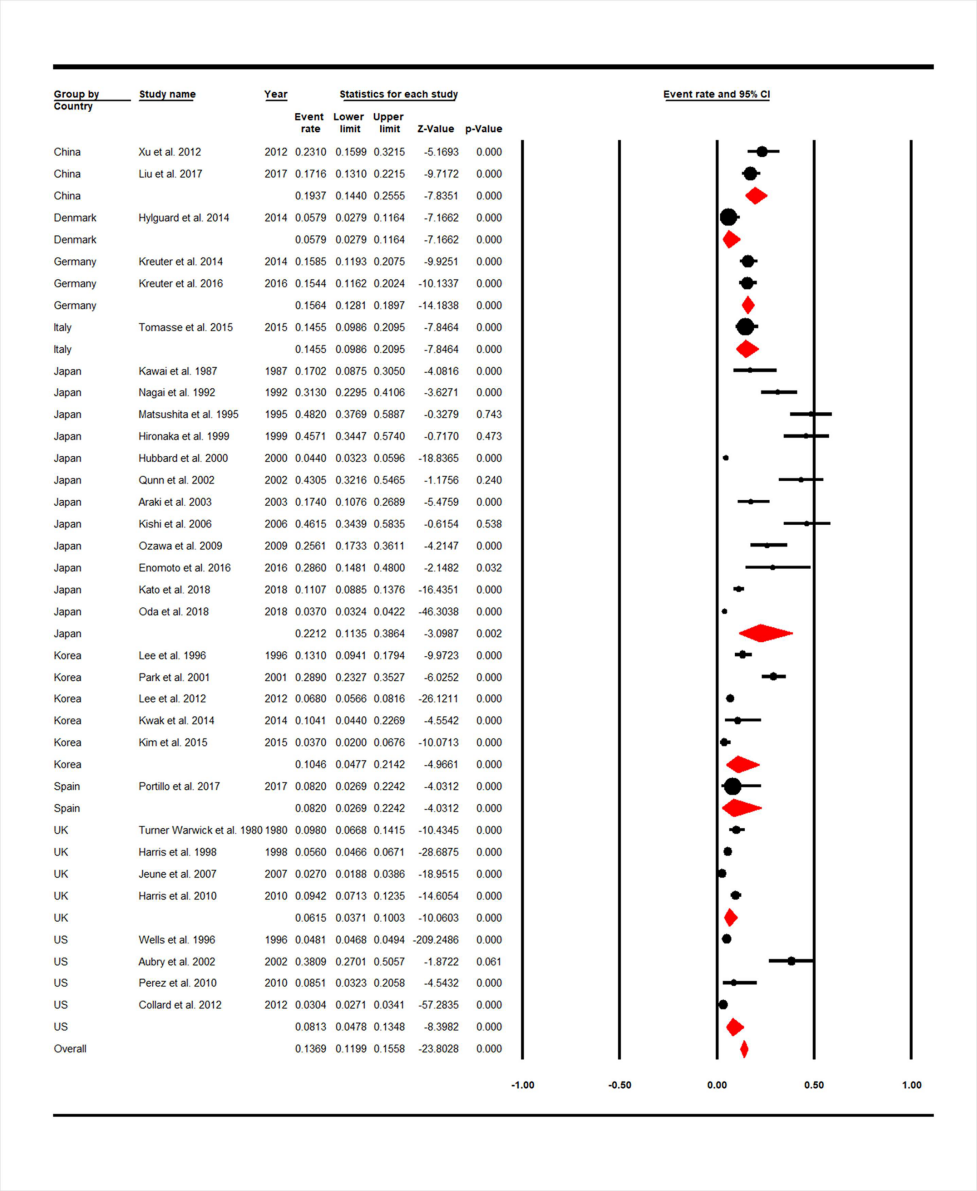
Forest plot for overall prevalence of LC in IPF sub-grouped based on country.

Among the studies in 8 countries, the highest and lowest prevalence of LC in IPF patients was in Japan with 12 studies and Denmark with 1 study that were estimated 22.12% (95% CI: 11.35–38.64) and 5.79% (95% CI: 2.79–11.64) which were statistically significant (P = 0.000) (Fig 3).

### 3.4. Cumulative meta-analysis and sensitivity analysis of studies

**Fig 4:**
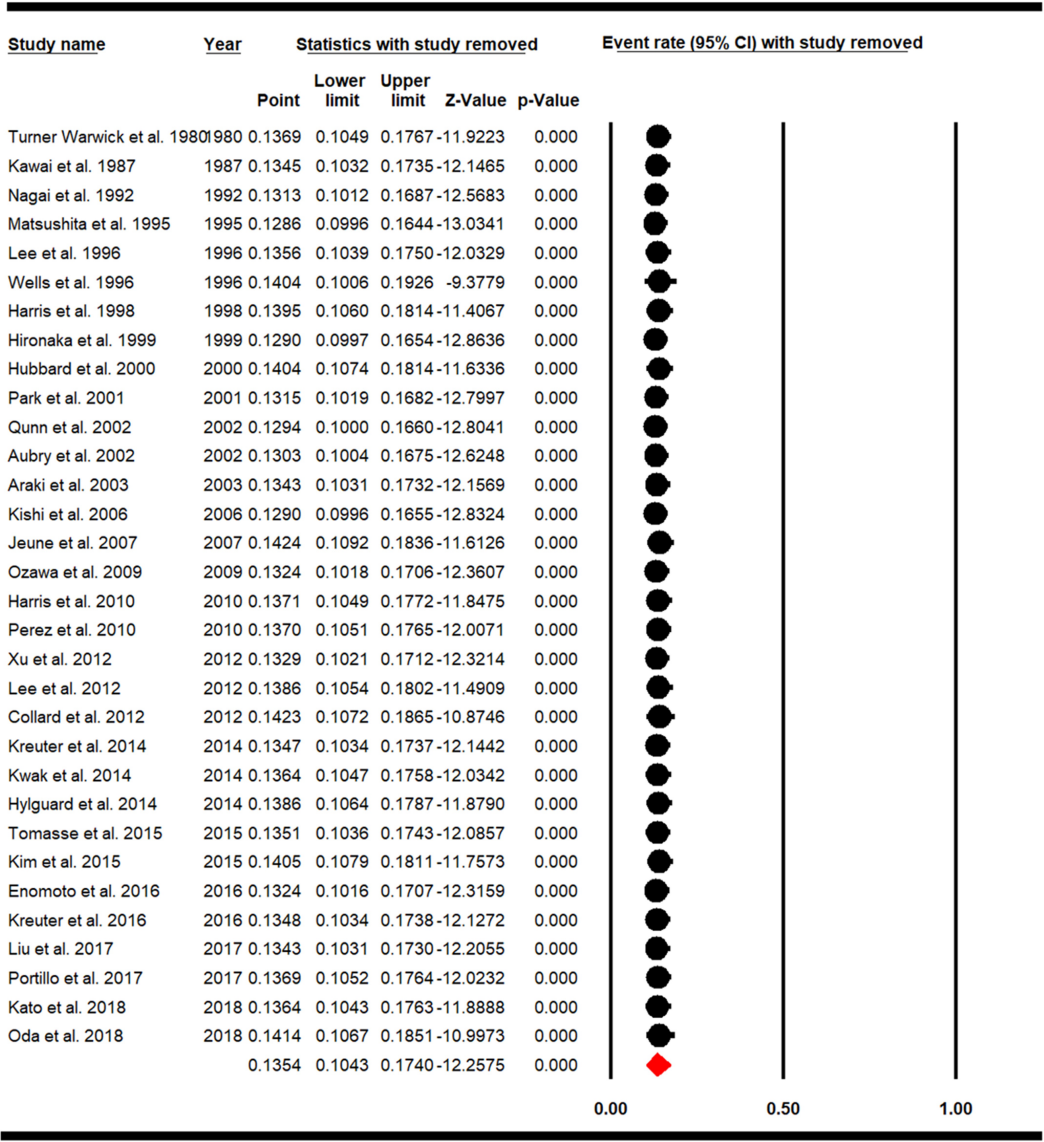
Sensitivity analysis for overall prevalence of LC in IPF.

**Fig 5:**
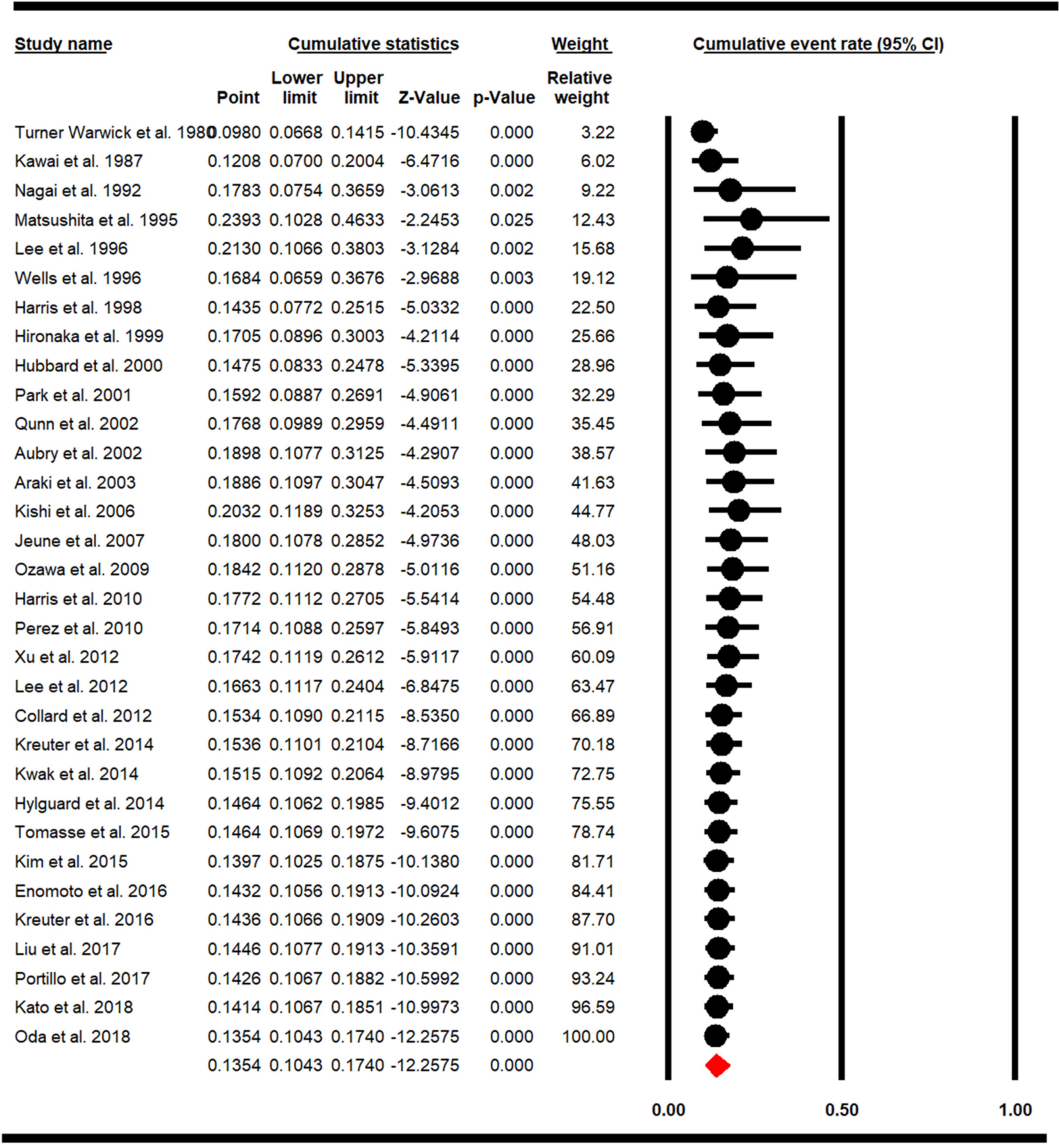
Cumulative meta-analysis for overall prevalence of LC in IPF.

Sensitivity analysis of LC prevalence in IPF patients and the confidence interval of each study was calculated with a 95% confidence interval, and results showed that before and after exclusion of each study there had been no significant effect on the overall prevalence rate of LC in IPF patients (Fig 4). Cumulative meta-analysis is also estimated for the overall prevalence of LC in IPF patients based on the publication year and is represented in (Fig 5).

### 3.5. Meta-regression

**Fig 6:**
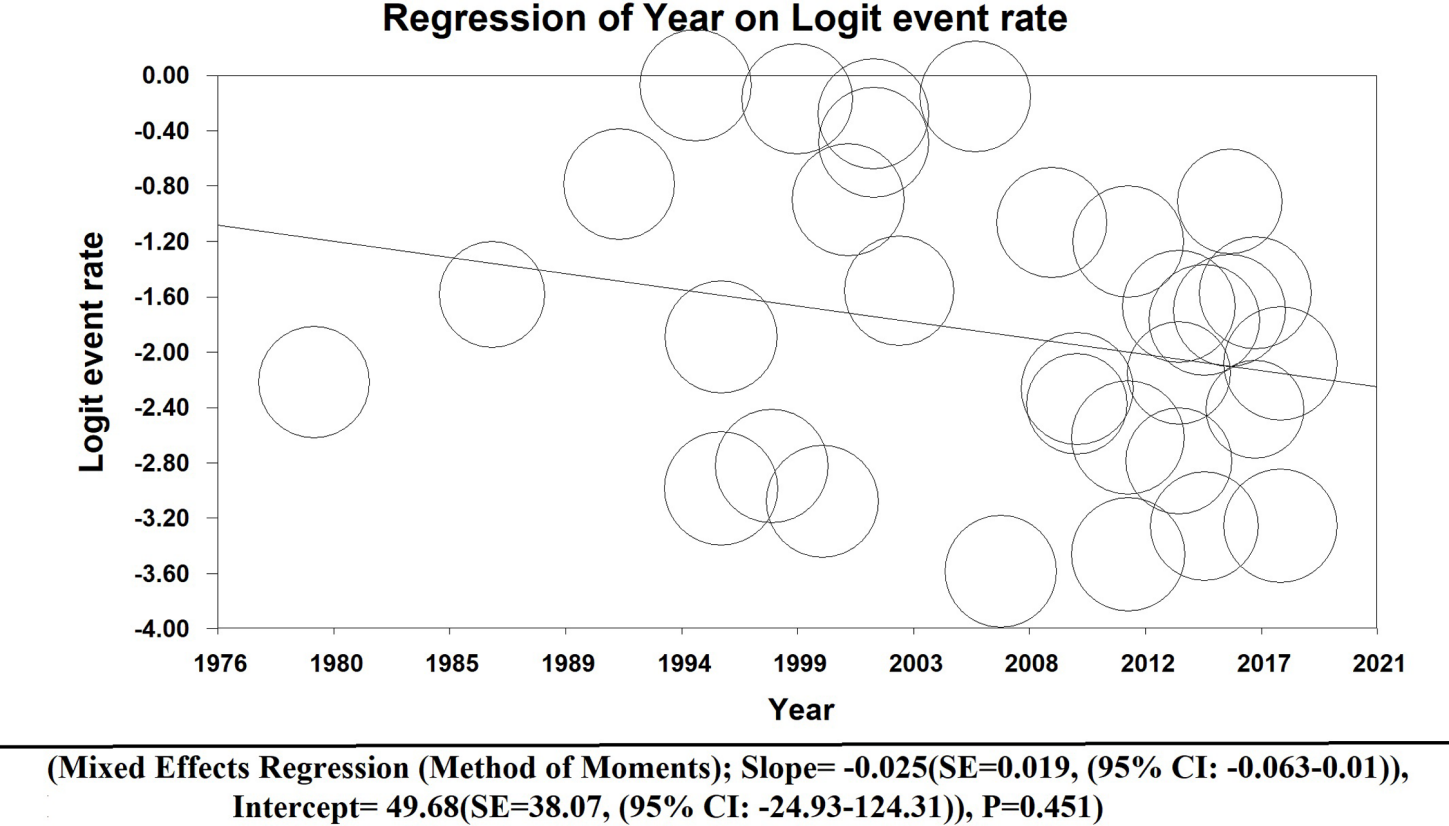
Meta-regression model of overall prevalence of LC in IPF (Method of moments).

The Meta-Regression test shows that the prevalence of LC in IPF patients has been decreased by the year of publication, but this relationship is not statistically significant (Mixed effects regression (Method of moments); Slope = -0.025(SE = 0.019, (95% CI: -0.063–0.01)), Intercept = 49.68(SE = 38.07, (95% CI: -24.93–124.31)), P = 0.451)(Fig 6).

### 3.6. Prevalence of LC in IPF based on gender

The prevalence rate of LC in IPF patients with female gender was estimated to be 9.48% (77 out of 890) (95% CI: 7.49–11.94) and in the male gender 91.22% (813 out of 890) (95% CI: 88.72–93.2) that this difference (1: 9; female: male) was statistically significant (P = 0.000) (S1 Fig).

### 3.7. Prevalence of smoking statue in LC-IPFs’ patients

The prevalence of smoking in LC-IPF patients were estimated to be 90.74% (95% CI: 87.07–93.44) (S2 Fig).

### 3.8. Cellular (histological) subtypes of LC in IPF

The prevalence of SQCC in IPF patients with LC was estimated 37.82% (95% CI: 32.65–43.29) (S3 Fig), which the highest prevalence was in France and the USA with 66.66% and the lowest was in China (27.17%). (S4 Fig). The prevalence of ADC in IPF patients with LC was estimated 30.79% (95% CI: 26.49–35.46) (S5 Fig), with the highest prevalence in China (41.58%), and the lowest in France (20%) (S6 Fig). The prevalence of SmCC in IPF patients with LC was estimated 20.48% (95% CI: 14.94–27.43) (S7 Fig), with the highest prevalence in Japan (24.93%) and the lowest prevalence in France (3.33%) (S8 Fig). The prevalence of LCC in IPF patients with LC was estimated 5.21% (95% CI: 2.91–9.17) (S9 Fig) that prevalence in Japan (6.21%) was estimated more than Korea (3.74%) (S10 Fig). The prevalence of ADQC in IPF patients with LC was estimated 4.81% (95% CI: 2.42–9.34) (S11 Fig.) that the highest prevalence was in China (8.3%) and the lowest was in Japan (3.15%) (S12 Fig).

### 3.9. Prevalence of LC in IPF according to clinical staging

The highest and lowest Stage of LC in IPF patients were estimated in III and II with a prevalence of 30.72% (95% CI: 22.68–40.14) and 13.33% (95% CI: 8.74–19.81), respectively (P = 0.000). The highest density was observed in Stage III and IV (S13 Fig).

### 3.10. Region and location of LC in IPF

The involvement location of LC in IPF patients was more pronounced in the peripheral area with a prevalence of 84.03% (95% CI: 75.47–89.99), which is statistically significant (P = 0.004) (S14 Fig). The tumor region involved in LC in IPF patients, from the highest to the lowest was estimated in the lower, upper and middle regions with a prevalence of 52.37% (95% CI: 47.54–57.15), 38.98% (95% CI: 33.2–45.05) and 5.37% (95% CI: 3.92–7.34), respectively, which is statistically significant (P = 0.000) (S15 Fig). The prevalence of the tumor region involved with the most detail from the highest to the lowest was estimated to be in the RLL, LLL, RUL and LUL regions with 32.74% (95% CI: 28.76–36.98), 23.46% (95% CI: 18.62–29.12), 19.99% (95% CI: 14.72–26.54) and 17.39% (95% CI: 14.26–21.03), respectively, which is statistically significant (P = 0.000) (S16 Fig).

### 3.11. Publication bias

**Fig 7:**
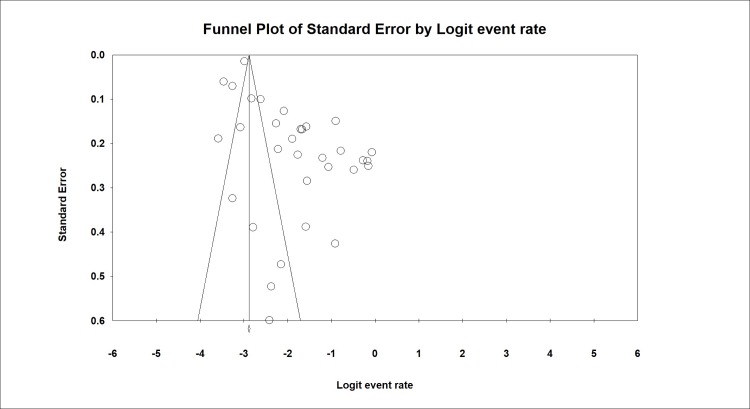
Publication bias of studies included due to the aim of prevalence of LC in IPF.

The publication bias was also evaluated by Begg and Egger’s tests and was estimated at P = 0.516 and P = 0.0521, respectively. In this test, the probability of publication bias was not statistically significant (Fig 7).

## 4. Discussion

The total sample size of patients with IPF was estimated 131947 among whom 6384 had LC. The total rate of LC prevalence in IPF patients based on the meta-analysis review was estimated to be 13.54% (95% CI: 10.43–17.4) that was significantly 9 times higher in men than women. Also, the prevalence of smoking in LC-IPF patients is estimated to be 9 times higher. Highest to lowest prevalence of cellular (histological) subtypes of lung cancer in IPF were SQCC (37.82%), ADC (30.79%), SmCC (20.48%), LCC (5.21%), and ADQC (4.81%), respectively. The highest and lowest stage of lung cancer in IPF patients was estimated at III and II, respectively. The highest involvement location of lung cancer in IPF patients was in the Peripheral region with highest to the lowest RLL, LLL, RUL and LUL regions respectively, that totally the highest to lowest was estimated to be in the lower, upper and middle regions.

Age and smoking status are also known to be the effective factors in the development of lung cancer in IPF patients [50, 70]. Nearly every patient with prostate cancer and lung cancer (95%) has finger clubbing, while the percentage of IPF patients are approximately only 60%, which is often known as the clinical evidence of lung cancer[40, 62].

Recent studies found that the progression of lung cancer in lower lobes is higher in IPF patients with[99, 100]. As also found in the findings of the present study, there is a significant relationship between the involved lobes, which can be a phenomenon called “scar–cinoma” between fibrotic areas and cancer progression[101]. Although further studies are required to prove this[101–105].

Evidence represent that IPF and LC have common pathological features[106]. Anticancer agents, like Nintedanib, show pleiotropic anti-fibrotic properties[28, 107, 108]. Among the common pathological characteristics of IPF and LC, the following can be mentioned: increased proliferation, proteus loss, immune dysregulation, senescence features and resistance against apoptosis, telomeric attrition, and disorder in bioenergetics of the *cell*[109–111]. Also, the key features of epithelial fibrosis are epithelial to mesenchymal transition (EMT), which plays an important role in lung cancer[101–105].

In another study, epigenetic and genetic changes, abnormal expression of microRNAs (miRNAs, cellular and molecular aberrances), like different responses to regulatory signals, apoptosis, delay or decrease in cell-to-cell correlation along with activation of specific signal transmission pathways lead to pathogenic features of both LC-IPF. Likewise, genetic analysis has shown that harmful and deleterious mutations in A1 or A2-surfactant proteins cause familial idiopathic interstitial pneumonia and lung cancer[112–114].

The heterogeneity rate (I^2^) in the present study was calculated at 90.71%, which is in the line of studies with high heterogeneity. It is assumed that the observed differences are due to various samplings and also the difference in the measured parameter in different societies[87, 88, 115].

According to the meta-regression, the prevalence of the LC-IPF was not statistically significant (p = 0.451) by the publication year. Even though the studies are in different countries and in years, but these findings cannot represent the reality in all countries, so further studies are needed to be conducted in this regard.

## 5. Limitations

One of the main limitations of this meta-analysis to be mentioned is the inclusion of studies with different inclusion and exclusion criteria, and there is no consensus definition of IPF expressed. Selection Bias is more discussed which can limit the generalization of these findings because the type of lung cancer in a country can be different with the other countries and could be related to descent diversities. Due to lack of resources and very few studies have investigated the survival rate and causes of mortality and the precise methods of treatment, according to the aim of the present study, we did not focus on these factors. Also, data were accessed by using Guilan University of Medical Sciences’ -Iran Ministry of Health & Medical Education- VPN which some databases are not fully accessible.

## 6. Conclusion

In conclusion, the high prevalence of the LC-IPF with 13.5% is more observed in older men who smoke, and is more evident in the progression of cancer, SQCC, and SmCC, and mostly affects the peripheral regions and the lower part of the lung. Studies have been conducted in limited countries, such as Japan, Korea, and UK and USA, which the weakness of a unit study of LC-IPF in different countries investigating the factors and important risk factors and reaching to a consensus and preparing a comprehensive global database for clinical decision-making is felt and is an essential need.

## Supporting information

S1 File PRISMA Checklist. (DOC)

S2 File The review protocol which has been registered in PROSPERO International Prospective Register of Systematic Reviews. (PDF)

S3 File Newcastle-Ottawa scale checklist. (PDF)

S1 Table The search strategy used for each database. (DOCX)

S2 Table Data characteristics (Full details) (MS Excel). (XLSX)

S1 Fig Prevalence of LC in IPF based on gender. (TIF)

S2 Fig Prevalence of smoking statue in LC-IPFs’ patients. (TIF)

S3 Fig The prevalence of SQCC in IPF patients with LC. (TIF)

S4 Fig The prevalence of SQCC in IPF patients with LC subgrouped by country. (TIF)

S5 Fig The prevalence of ADC in IPF patients with LC. (TIF)

S6 Fig The prevalence of ADC in IPF patients with LC subgrouped by country. (TIF)

S7 Fig The prevalence of SmCC in IPF patients with LC. (TIF)

S8 Fig The prevalence of SmCC in IPF patients with LC subgrouped by country. (TIF)

S9 Fig The prevalence of LCC in IPF patients with LC. (TIF)

S10 Fig The prevalence of LCC in IPF patients with LC subgrouped by country. (TIF)

S11 Fig The prevalence of ADQC in IPF patients with LC. (TIF)

S12 Fig The prevalence of ADQC in IPF patients with LC subgrouped by country. (TIF)

S13 Fig Prevalence of LC in IPF according to clinical staging. (TIF)

S14 Fig Location of LC in IPF patients. (TIF)

S15 Fig Region (Upper/Lower/Middle) of LC in IPF. (TIF)

S16 Fig Region (LLL/LUL/RLL/RUL) of LC in IPF. (TIF)
