# Supplementary material for: Lung cancer in idiopathic pulmonary fibrosis: A systematic review and meta-analysis
Source: PLoS One. 2018 Aug 16;13(8):e0202360. doi: 10.1371/journal.pone.0202360 (PMC6095562; doi:10.1371/journal.pone.0202360)
Supplement: S2 File — (PDF) [file pone.0202360.s003.pdf]

Lung cancer in idiopathic pulmonary fibrosis: a systematic review and meta-analysis

Mohammad Hossein YektaKooshali, AliReza JafariNezhad

Citation

Mohammad Hossein YektaKooshali, AliReza JafariNezhad. Lung cancer in idiopathic pulmonary fibrosis: a systematic review and meta-analysis. PROSPERO 2018 CRD42018094037 Available from: http://www.crd.york.ac.uk/PROSPERO/display_record.php?ID=CRD42018094037

Review question

What is the overall prevalence of lung cancer (LC) in idiopathic pulmonary fibrosis (IPF)?
What is the prevalence of LC in IPF based on country?
What is the prevalence of LC in IPF based on gender?
What is the prevalence of smoking in LC-IPF patients?

What is the prevalence of the cellular (histological) subtypes of LC in IPF ((ADC (adenocarcinoma (%)), SQCC (squamous-cell carcinoma (%)), SmCC (small-cell carcinoma (%)), LCC (large-cell carcinoma (%)), ADSQC (adeno-squamous carcinoma (%)), and others)?

What is the prevalence of LC in IPF according to clinical staging (I, II, II, IV)?

What is the prevalence of the region and location of LC in IPF (region: RUL (right upper lobe), LUL (left upper lobe), RLL (right lower lobe), LLL (left lower lobe), upper, center, lower, and location: peripheral and central)?

Searches

The present study will be carried out in accordance with the Meta-analysis of Observational Studies in Epidemiology guideline, and according to the PRISMA statement.
Search strategy:
A related search was conducted using international databases, such as Web of Science, Scopus, PubMed/MEDLINE, OVID, EMBASE, and The Cochrane Library, to collect all available studies on LC in IPF patients.
The searches were conducted using standard keywords as well as MeSH and MeSH Entry, and all probabilistic combinations of words using Boolean operators combined on the databases without time limits, up to 03/25/2018.
In addition, a manual search was also carried out of the reference lists of relevant articles.
Importance was placed on searching the databases using a high-sensitivity strategy, and the search was conducted by the researcher and a senior specialist familiar with searching the databases (A.R).

Types of study to be included

Retrospective studies.

Condition or domain being studied

Lung cancer in idiopathic pulmonary fibrosis.

Participants/population

Cohort and retrospective studies which have investigated LC in IPF patients.

Intervention(s), exposure(s)

Surgical resection of radiologically- and pathologically-confirmed cancer.

Comparator(s)/control

Prevalence of LC in patients with IPF will be compared with rates of non-occurrence of LC.

Context

Primary outcome(s)

Overall prevalence of lung cancer (LC) in idiopathic pulmonary fibrosis (IPF).

Secondary outcome(s)

Prevalence of LC in IPF by country;

Prevalence of LC in IPF by gender;
Prevalence of smoking in LC-IPF patients;

Prevalence of the cellular (histological) subtypes of LC in IPF ((ADC (adenocarcinoma (%)), SQCC (squamous-cell carcinoma (%)), SmCC (small-cell carcinoma (%)), LCC (large-cell carcinoma (%)), ADSQC (adeno-squamous carcinoma (%)), and others);

Prevalence of LC in IPF according to clinical staging (I, II, II, IV);

Prevalence of the region and location of LC in IPF (region: RUL (right upper lobe), LUL (left upper lobe), RLL (right lower lobe), LLL (left lower lobe), upper, center, lower, and location: peripheral and central).

Data extraction (selection and coding)

The following information will be extracted and entered into an Excel spreadsheet:
authors, year, place, sample size (SS), periods of time, SS of IPF, SS of LC, prevalence (LC in IPF), cellular (histological) subtypes, clinical staging, prevalence by gender, prevalence by smoking status, FEV1 (forced expiratory volume in the first second) (mean±SD), FVC1 (forced vital capacity in the first second) (mean±SD), FEV1/FVC1 (mean±SD), age (mean±SD), DLco (diffusing capacity for carbon monoxide) (mean±SD), region and location.

Risk of bias (quality) assessment

After eliminating irrelevant studies, the quality of the final studies will be examined. The Newcastle-Ottawa Scale (NOS) checklist will be used, which consists of eight sections, and divides the studies with a scale of scores of 0 to 8 from poor to high quality, respectively. The studies are also divided into three levels of scoring:
1- Studies with a score of 5 or less: poor quality;
2- Studies with a score of 5-6: medium quality;
3- Studies with a score of 7 to 8: high quality.
Articles of medium to high quality will be used in the data analysis.

Strategy for data synthesis

The present study will be carried out in accordance with the Meta-analysis of Observational Studies in Epidemiology guideline, and according to the PRISMA statement, and will be based on five steps, including the design and search strategy, the collection of articles and their systematic review, the evaluation of the inclusion and exclusion criteria, and the qualitative evaluation and statistical analysis of the data. All stages of the research will be carried out by two researchers independently and, in the event of any differences in opinion, a specialist will carefully investigate the issue.

We will provide a narrative synthesis of the results from the included studies.
The prevalence rate of LC in IPF patients will be considered based on binomial distribution probability, and its variance will be calculated by binomial distribution, the Cochran test (Q) and I^2 index will be used for the evaluation of heterogeneity. An I^2 index less than 25% is indicative of low heterogeneity, between 75% -25% represents average heterogeneity, and more than 75% means that considerable heterogeneity is present. Sensitivity analysis (one study removed) will also be conducted to investigate the impact of each study on

the total results of the overall prevalence, and the assessment of each of the risk factors.
In order to evaluate the causes of any heterogeneity, subgroup analyses will be performed based on the country, the quality of the studies, and the gender of the participants, and a meta-regression model will be used to determine prevalence rates based on the year of publication.
The Begg's and Egger's test will be applied using a funnel plot to examine the possible existence of publication bias.
Data analysis will be analyzing using Comprehensive Meta-Analysis Ver. 2, and the significance level of the test will be < 0.05.

Analysis of subgroups or subsets

The following subgroup analyses will be carried out:
Prevalence of LC in IPF based on gender; country,
Prevalence of smoking in LC-IPF patients;
Cellular (histological) subtypes of LC in IPF;
Prevalence of LC in IPF according to clinical staging (I, II, II, IV);
Prevalence of region and location of LC in IPF.

Contact details for further information

Mohammad Hossein YektaKooshali
Yektakooshali.mh1995@yahoo.co.uk

Organisational affiliation of the review

Guilan University of Medical Sciences, Rasht, Iran

Review team members and their organisational affiliations

Dr Mohammad Hossein YektaKooshali. School of nursing, Midwifery and Paramedicine, Guilan University of Medical Sciences, Rasht, Iran
Assistant/Associate Professor AliReza JafariNezhad. Inflammatory Lung Disease Research Center, Department of Internal Medicine, Razi Hospital, School of Medicine, Guilan University of Medical Sciences, Rasht, Iran

Collaborators

Mr Aboozar Ramezani. Ph.D. in Medical Library and Information, Department of Medical Library and Information, Virtual School, Tehran University of Medical Sciences, Tehran, Iran

Anticipated or actual start date

25 March 2018

Anticipated completion date

30 May 2018

Funding sources/sponsors

None

Conflicts of interest

None known.

Language

English

Country

Iran

Stage of review

Review_Ongoing

Subject index terms status

Subject indexing assigned by CRD

Subject index terms

Humans; Idiopathic Pulmonary Fibrosis; Incidence; Lung; Lung Neoplasms; Prevalence; Risk Factors

Date of registration in PROSPERO

30 April 2018

Date of publication of this version

30 April 2018

Details of any existing review of the same topic by the same authors

Stage of review at time of this submission

Stage	Started	Completed
Preliminary searches	Yes	Yes
Piloting of the study selection process	Yes	No
Formal screening of search results against eligibility criteria	Yes	No
Data extraction	No	No
Risk of bias (quality) assessment	No	No
Data analysis	No	No

Versions

30 April 2018

PROSPERO
This information has been provided by the named contact for this review. CRD has accepted this information in good faith and registered the review in PROSPERO. CRD bears no responsibility or liability for the content of this registration record, any associated files or external websites.
