# Supplementary material for: Lung cancer in idiopathic pulmonary fibrosis: A systematic review and meta-analysis
Source: PLoS One. 2018 Aug 16;13(8):e0202360. doi: 10.1371/journal.pone.0202360 (PMC6095562; doi:10.1371/journal.pone.0202360)
Supplement: S3 File — (PDF) [file pone.0202360.s004.pdf]

Newcastle-Ottawa Quality Assessment Form for Cohort Studies

Note: A study can be given a maximum of one star for each numbered item within the Selection and Outcome categories. A maximum of two stars can be given for Comparability.

Selection

1) Representativeness of the exposed cohort
   a) Truly representative ***(one star)***
   b) Somewhat representative ***(one star)***
   c) Selected group
   d) No description of the derivation of the cohort
2) Selection of the non-exposed cohort
   a) Drawn from the same community as the exposed cohort ***(one star)***
   b) Drawn from a different source
   c) No description of the derivation of the non exposed cohort
3) Ascertainment of exposure
   a) Secure record (e.g., surgical record) ***(one star)***
   b) Structured interview ***(one star)***
   c) Written self report
   d) No description
   e) Other
4) Demonstration that outcome of interest was not present at start of study
   a) Yes ***(one star)***
   b) No

Comparability

1) Comparability of cohorts on the basis of the design or analysis controlled for confounders
   a) The study controls for age, sex and marital status ***(one star)***
   b) Study controls for other factors (list) _________________________________ ***(one star)***
   c) Cohorts are not comparable on the basis of the design or analysis controlled for confounders

Outcome

1) Assessment of outcome
   a) Independent blind assessment ***(one star)***
   b) Record linkage ***(one star)***
   c) Self report
   d) No description
   e) Other
2) Was follow-up long enough for outcomes to occur
   a) Yes ***(one star)***
   b) No

Indicate the median duration of follow-up and a brief rationale for the assessment above:____________________

3) Adequacy of follow-up of cohorts
   a) Complete follow up- all subject accounted for ***(one star)***
   b) Subjects lost to follow up unlikely to introduce bias- number lost less than or equal to 20% or description of those lost suggested no different from those followed. ***(one star)***
   c) Follow up rate less than 80% and no description of those lost
   d) No statement
