# Supplementary material for: Lung cancer in idiopathic pulmonary fibrosis: A systematic review and meta-analysis
Source: PLoS One. 2018 Aug 16;13(8):e0202360. doi: 10.1371/journal.pone.0202360 (PMC6095562; doi:10.1371/journal.pone.0202360)
Supplement: S1 Table — (DOCX) [file pone.0202360.s002.DOCX]

**S1 Table. Search strategy used for one of the databases**

(Accessed by using Guilan University of Medical sciences’ -Iran Ministry of Health & Medical Education- VPN)

| **Databases** | **Group** | **Search Syntax** | **Records** |
| --- | --- | --- | --- |
| PubMed/Medline | 1 | (“Idiopathic Pulmonary Fibrosis”[MeSH Terms] OR “Idiopathic Pulmonary Fibroses”[Title/Abstract] OR “Familial Idiopathic Pulmonary Fibrosis”[Title/Abstract] OR “Fibrocystic Pulmonary Dysplasia”[Title/Abstract] OR “Fibrocystic Pulmonary Dysplasias”[Title/Abstract] OR “Hamman-Rich Disease”[Title/Abstract] OR “Hamman Rich Disease”[Title/Abstract] OR “Hamman-Rich Diseases”[Title/Abstract] OR “Usual Interstitial Pneumonitides”[Title/Abstract] OR “Usual Interstitial Pneumonitis”[Title/Abstract] OR “Usual Interstitial Pneumonia”[Title/Abstract] OR “Usual Interstitial Pneumonias”[Title/Abstract] OR “Cryptogenic Fibrosing Alveolitis”[Title/Abstract] OR “Cryptogenic Fibrosing Alveolitides”[Title/Abstract] OR “IPF”[Title/Abstract])) | **6521** |
|  | 2 | (“Lung Neoplasms”[MeSH Terms] OR “Pulmonary Neoplasms”[Title/Abstract] OR “Lung Neoplasm”[Title/Abstract] OR “Pulmonary Neoplasm”[Title/Abstract] OR “Lung Cancer”[Title/Abstract] OR “Lung Cancers”[Title/Abstract] OR “Pulmonary Cancer”[Title/Abstract] OR “Pulmonary Cancers”[Title/Abstract] OR “Cancer of the Lung”[Title/Abstract] OR “Cancer of Lung”[Title/Abstract]) | **247858** |
|  | **#1 AND #2** | | **385** |
| Scopus | 1 | TITLE-ABS-KEY(“Idiopathic Pulmonary Fibrosis” OR “Idiopathic Pulmonary Fibroses” OR “Familial Idiopathic Pulmonary Fibrosis” OR “Fibrocystic Pulmonary Dysplasia” OR “Fibrocystic Pulmonary Dysplasias” OR “Hamman-Rich Disease” OR “Hamman Rich Disease” OR “Hamman-Rich Diseases” OR “Usual Interstitial Pneumonitides” OR “Usual Interstitial Pneumonitis” OR “Usual Interstitial Pneumonia” OR “Usual Interstitial Pneumonias” OR “Cryptogenic Fibrosing Alveolitis” OR “Cryptogenic Fibrosing Alveolitides” OR “IPF”) | **10612** |
|  | 2 | TITLE-ABS-KEY (“Lung Neoplasms” OR “Pulmonary Neoplasms” OR “Lung Neoplasm” OR “Pulmonary Neoplasm” OR “Lung Cancer” OR “Lung Cancers” OR “Pulmonary Cancer” OR “Pulmonary Cancers” OR “Cancer of the Lung” OR “Cancer of Lung”) | **291013** |
|  | **#1 AND #2** | | **671** |
| Web of Sciences | 1 | **TOPIC:**((“Idiopathic Pulmonary Fibrosis” OR “Idiopathic Pulmonary Fibroses” OR “Familial Idiopathic Pulmonary Fibrosis” OR “Fibrocystic Pulmonary Dysplasia” OR “Fibrocystic Pulmonary Dysplasias” OR “Hamman-Rich Disease” OR “Hamman Rich Disease” OR “Hamman-Rich Diseases” OR “Usual Interstitial Pneumonitides” OR “Usual Interstitial Pneumonitis” OR “Usual Interstitial Pneumonia” OR “Usual Interstitial Pneumonias” OR “Cryptogenic Fibrosing Alveolitis” OR “Cryptogenic Fibrosing Alveolitides” OR “IPF”)) | **13170** |
|  | 2 | **TOPIC:** ((“Lung Neoplasms” OR “Pulmonary Neoplasms” OR “Lung Neoplasm” OR “Pulmonary Neoplasm” OR “Lung Cancer” OR “Lung Cancers” OR “Pulmonary Cancer” OR “Pulmonary Cancers” OR “Cancer of the Lung” OR “Cancer of Lung”)) | **219092** |
|  | **#1 AND #2** | | **676** |
| OVID | 1 | Idiopathic Pulmonary Fibrosis.sh. or Idiopathic Pulmonary Fibroses.af. or Familial Idiopathic Pulmonary Fibrosis.af. or Fibrocystic Pulmonary Dysplasia.af. or Fibrocystic Pulmonary Dysplasias.af. or Hamman-Rich Disease.af. or Hamman Rich Disease.af. or Hamman-Rich Diseases.af. or Usual Interstitial Pneumonitides.af. or Usual Interstitial Pneumonitis.af. or Usual Interstitial Pneumonia.af. or Usual Interstitial Pneumonias.af. or Cryptogenic Fibrosing Alveolitis.af. or Cryptogenic Fibrosing Alveolitides.af. or IPF.af. | **17088** |
|  | 2 | Lung Neoplasms.sh. or Pulmonary Neoplasms.af. or Lung Neoplasm.af. or Pulmonary Neoplasm.af. or Lung Cancer.af. or Lung Cancers.af. or Pulmonary Cancer.af. or Pulmonary Cancers.af. or Cancer of the Lung.af. or Cancer of Lung.af. | **472357** |
|  | **#1 AND #2** | | **17088** |
| COCHRANE |  | '("Idiopathic Pulmonary Fibrosis" OR "Idiopathic Pulmonary Fibroses" OR "Familial Idiopathic Pulmonary Fibrosis" OR "Fibrocystic Pulmonary Dysplasia" OR "Fibrocystic Pulmonary Dysplasias" OR "Hamman-Rich Disease" OR "Haman Rich Disease" OR "Hamman-Rich Diseases" OR "Usual Interstitial Pneumonitides" OR "Usual Interstitial Pneumonitis" OR "Usual Interstitial Pneumonia" OR "Usual Interstitial Pneumonias" OR "Cryptogenic Fibrosing Alveolitis" OR "Cryptogenic Fibrosing Alveolitides" OR "IPF") in Title, Abstract, Keywords in Trials and Other Reviews and Cochrane Reviews' | **634** |
|  |  | '("Lung Neoplasms" OR "Pulmonary Neoplasms" OR "Lung Neoplasm" OR "Pulmonary Neoplasm" OR "Lung Cancer" OR "Lung Cancers" OR "Pulmonary Cancer" OR "Pulmonary Cancers" OR "Cancer of the Lung" OR "Cancer of Lung") in Title, Abstract, Keywords in Trials and Other Reviews and Cochrane Reviews' | **13381** |
|  | **#1 AND #2** | | **10** |
