# Supplementary figures and images for: Lung cancer in idiopathic pulmonary fibrosis: A systematic review and meta-analysis
Source: PLoS One. 2018 Aug 16;13(8):e0202360. doi: 10.1371/journal.pone.0202360 (PMC6095562; doi:10.1371/journal.pone.0202360)

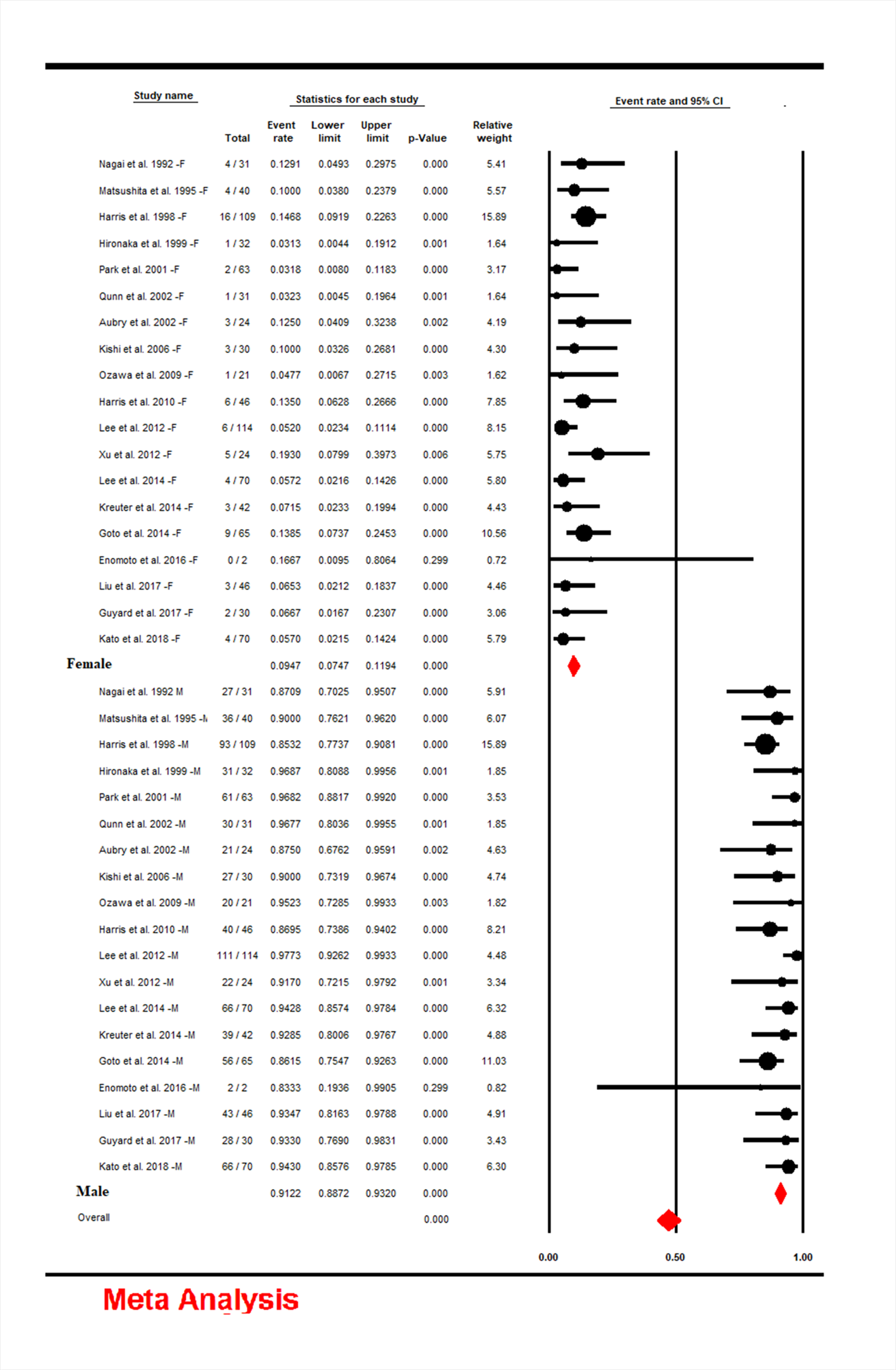

Supplement: S1 Fig — (TIF) [file pone.0202360.s006.tif]

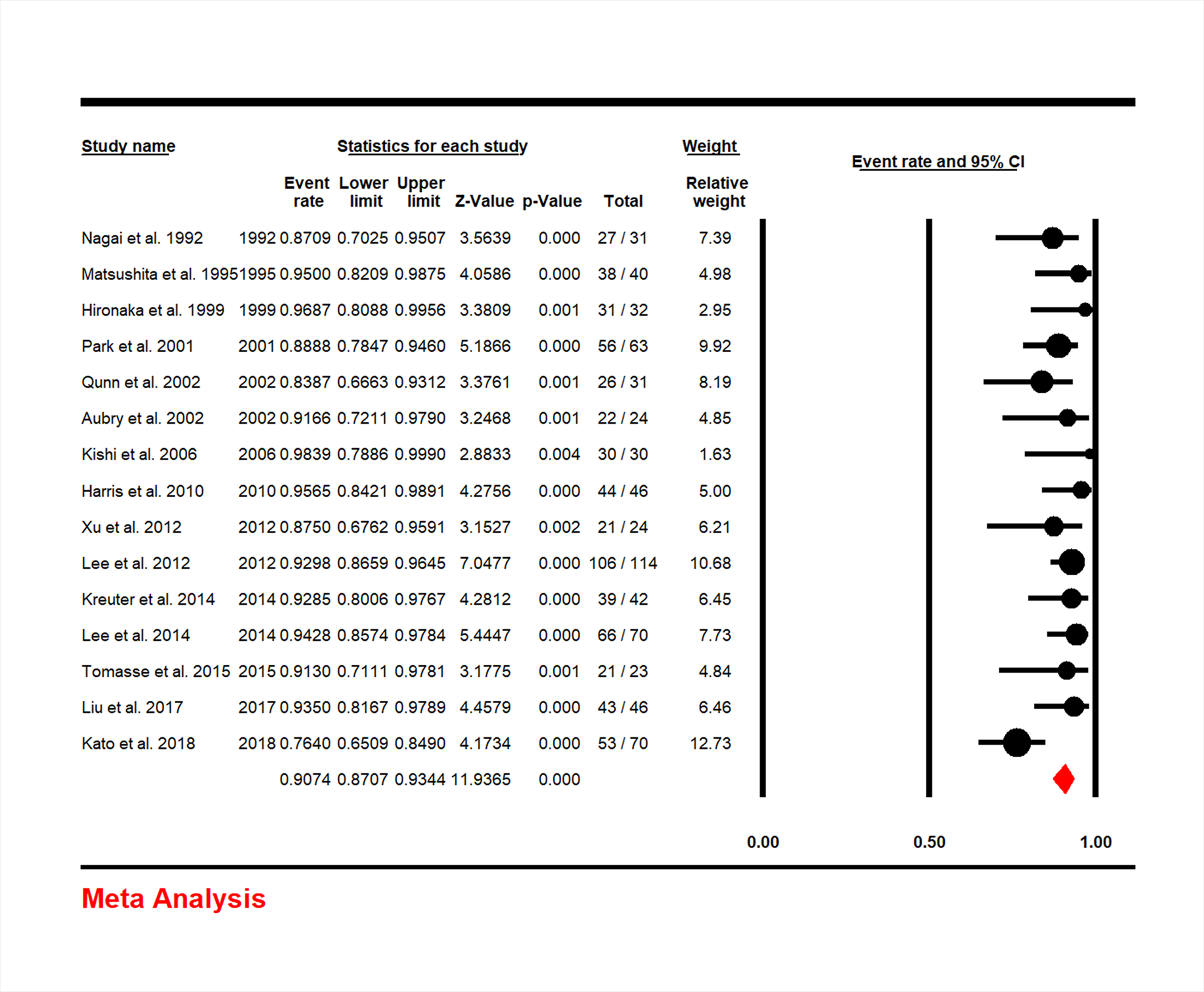

Supplement: S2 Fig — (TIF) [file pone.0202360.s007.tif]

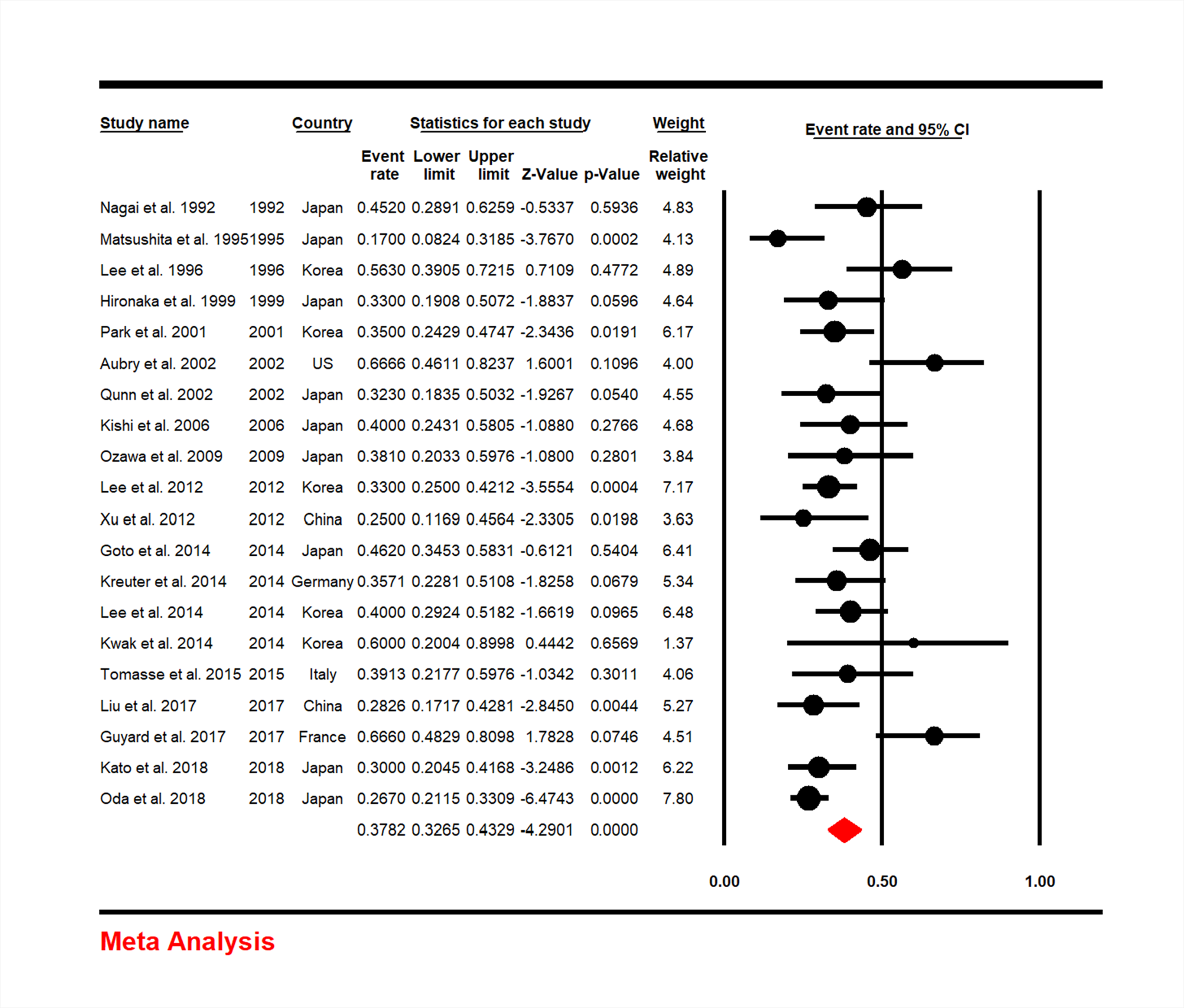

Supplement: S3 Fig — (TIF) [file pone.0202360.s008.tif]

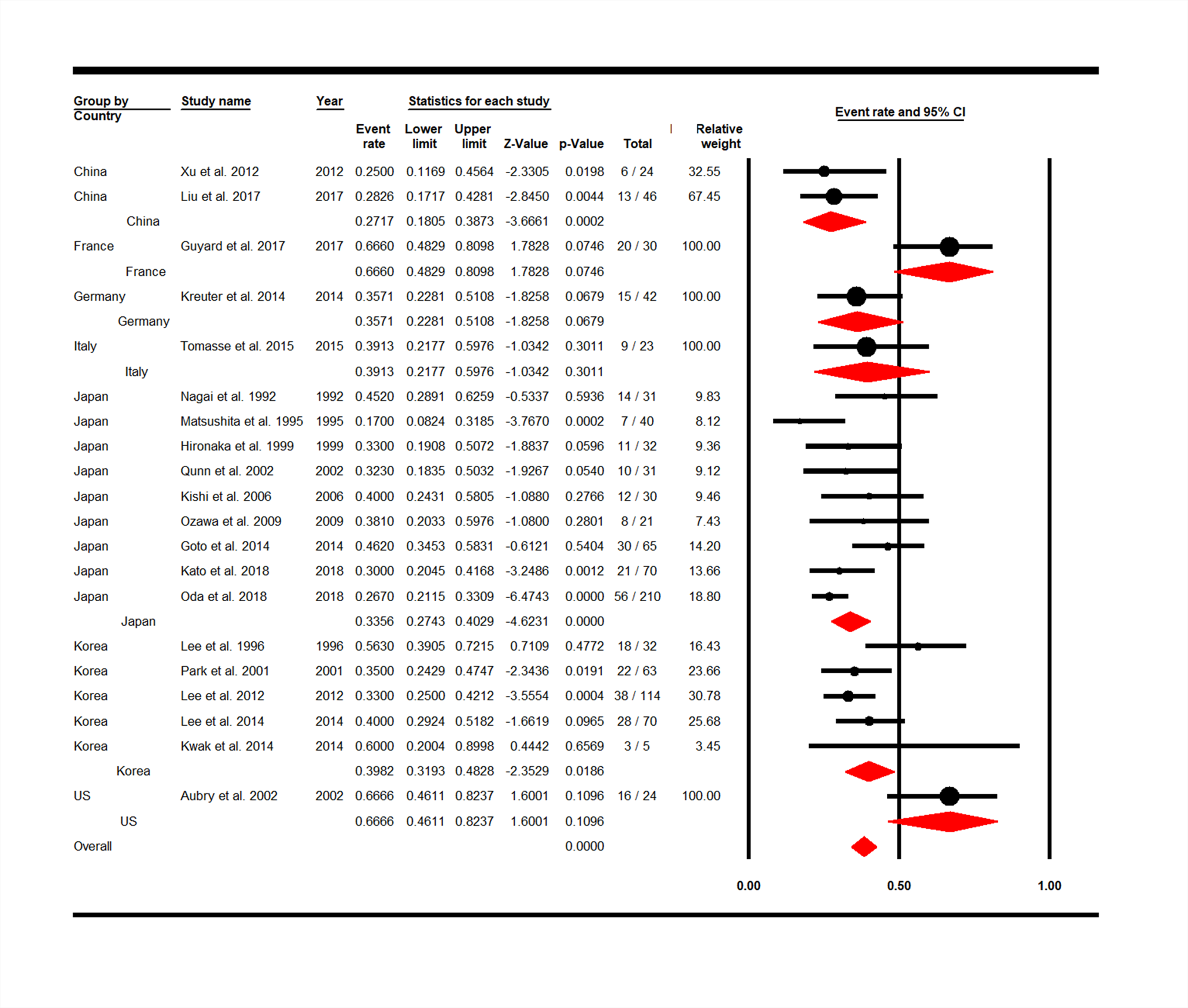

Supplement: S4 Fig — (TIF) [file pone.0202360.s009.tif]

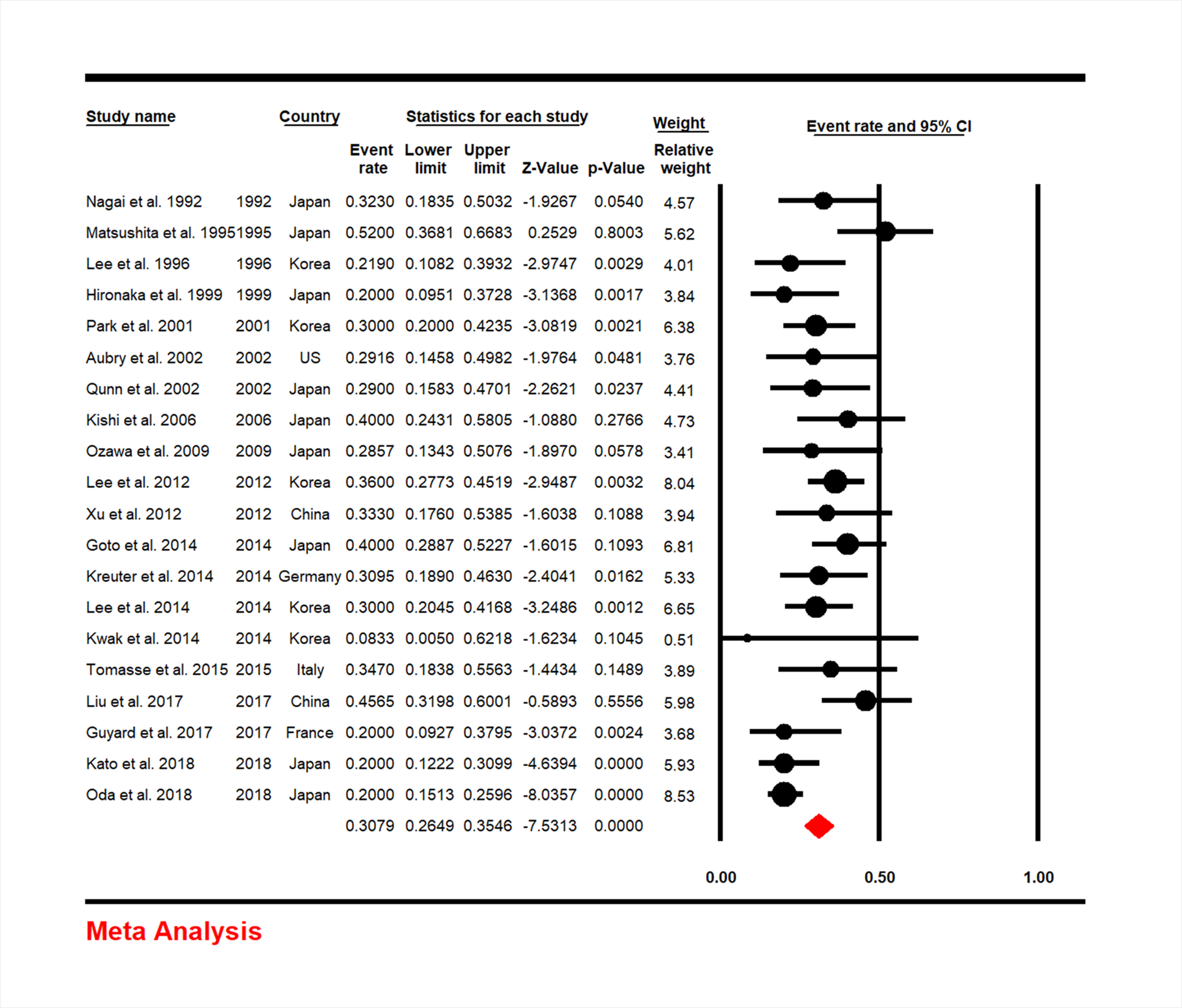

Supplement: S5 Fig — (TIF) [file pone.0202360.s010.tif]

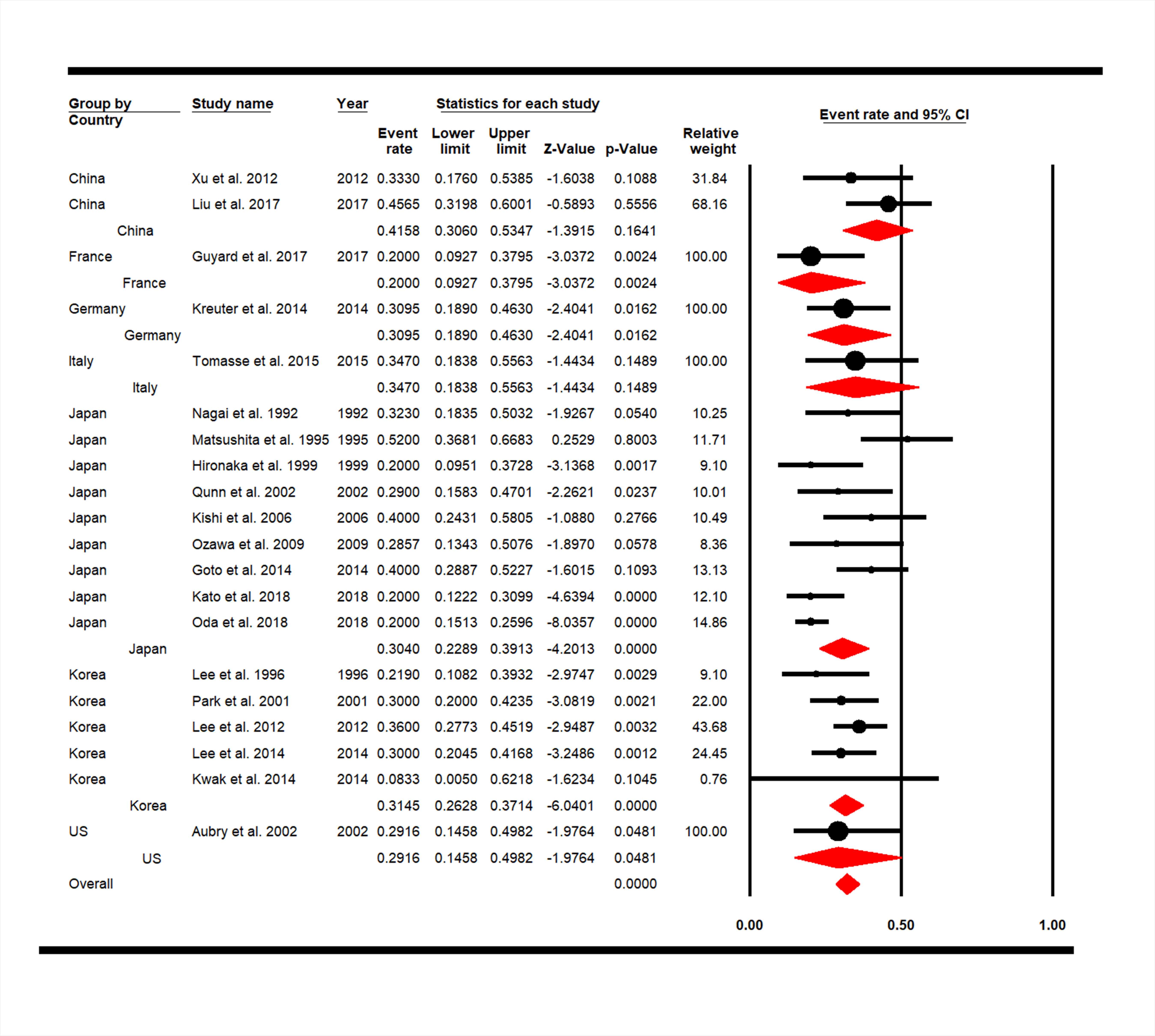

Supplement: S6 Fig — (TIF) [file pone.0202360.s011.tif]

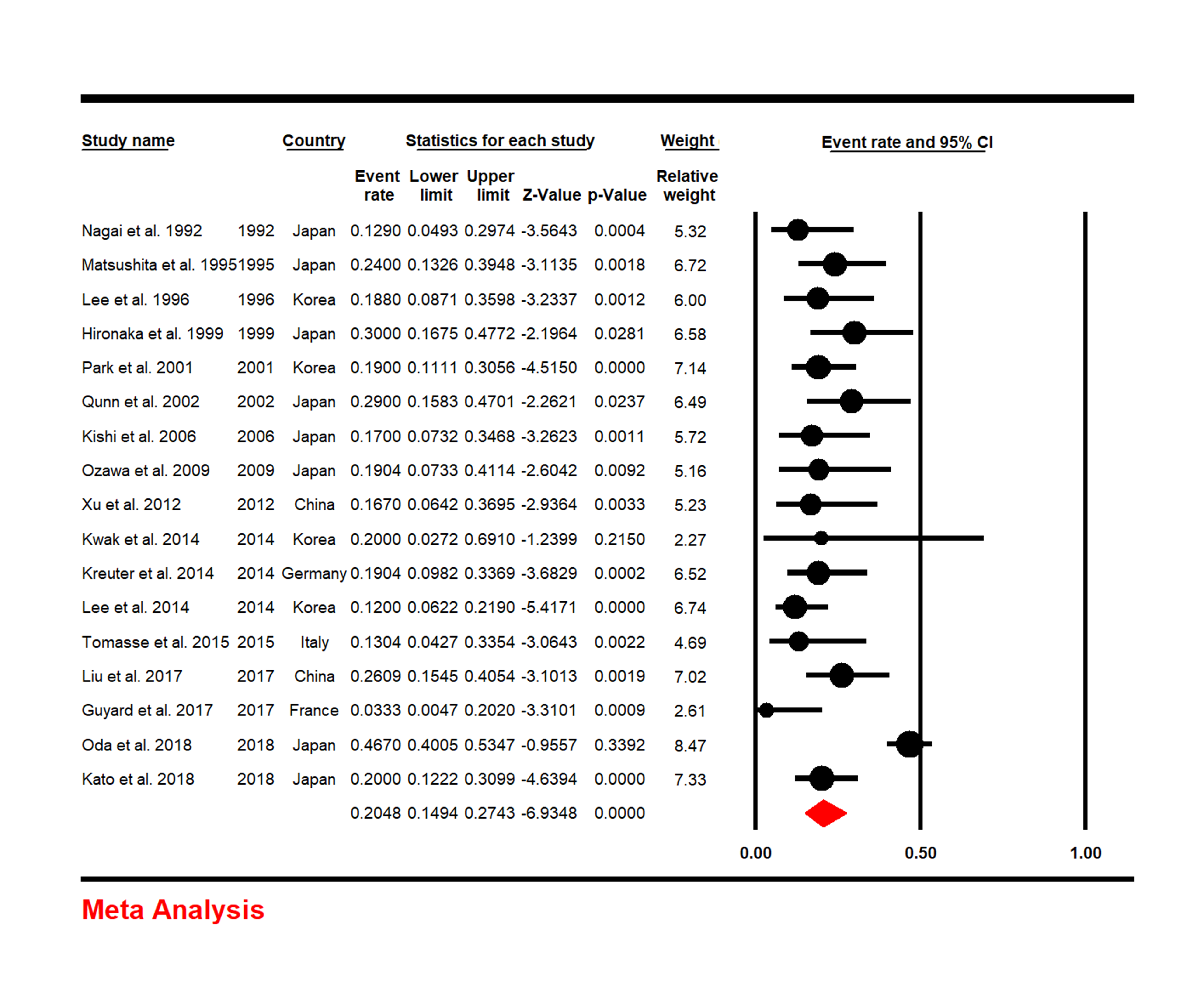

Supplement: S7 Fig — (TIF) [file pone.0202360.s012.tif]

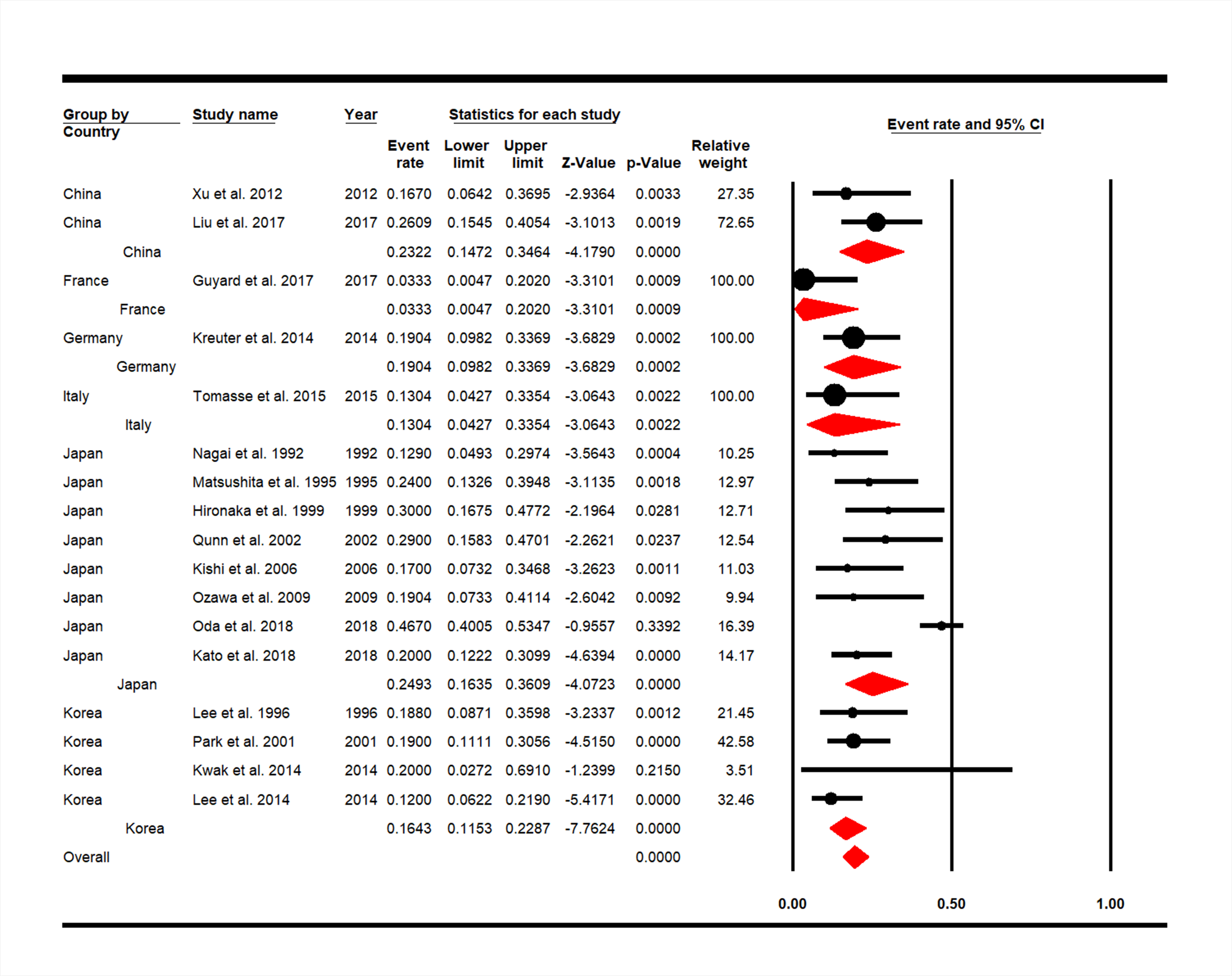

Supplement: S8 Fig — (TIF) [file pone.0202360.s013.tif]

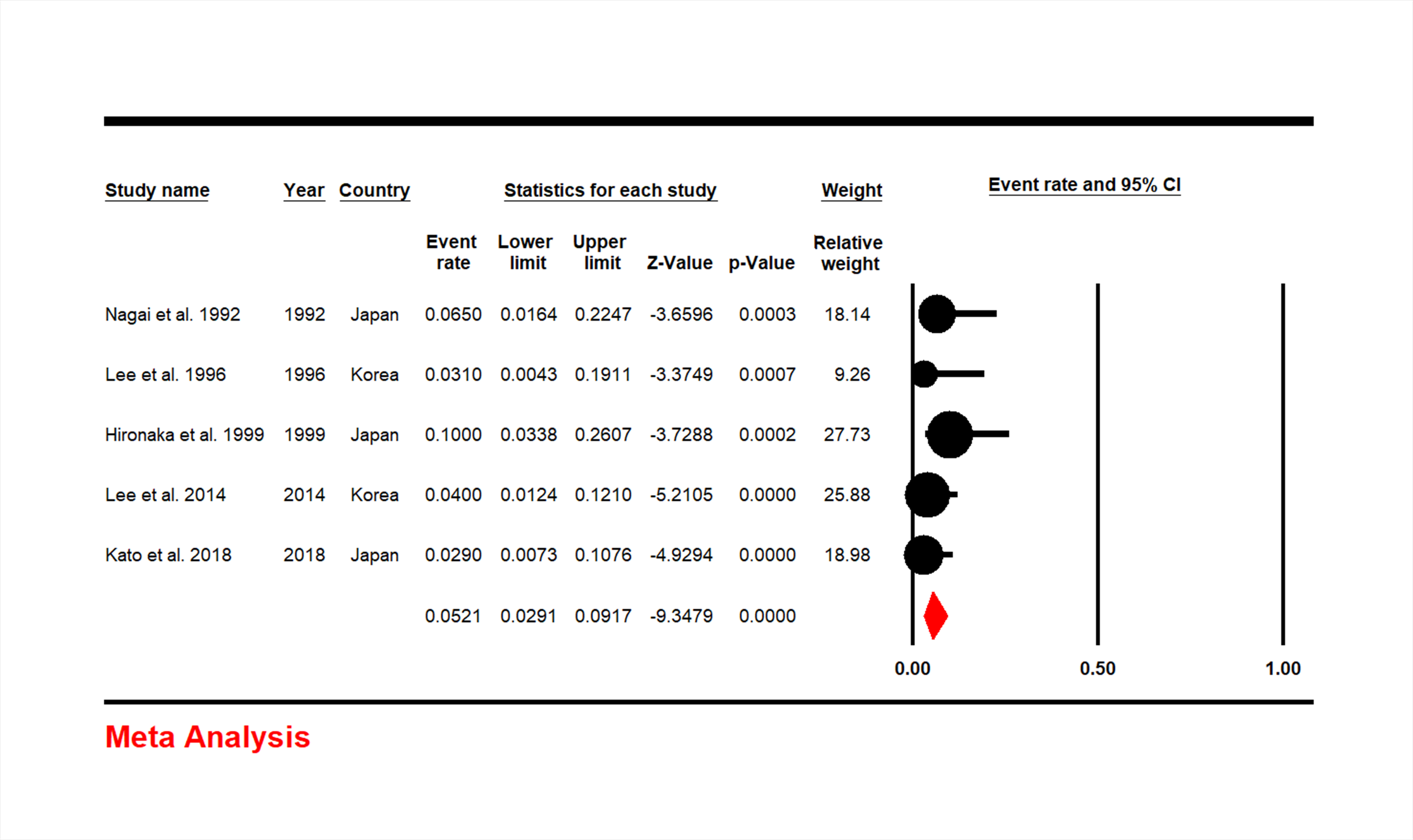

Supplement: S9 Fig — (TIF) [file pone.0202360.s014.tif]

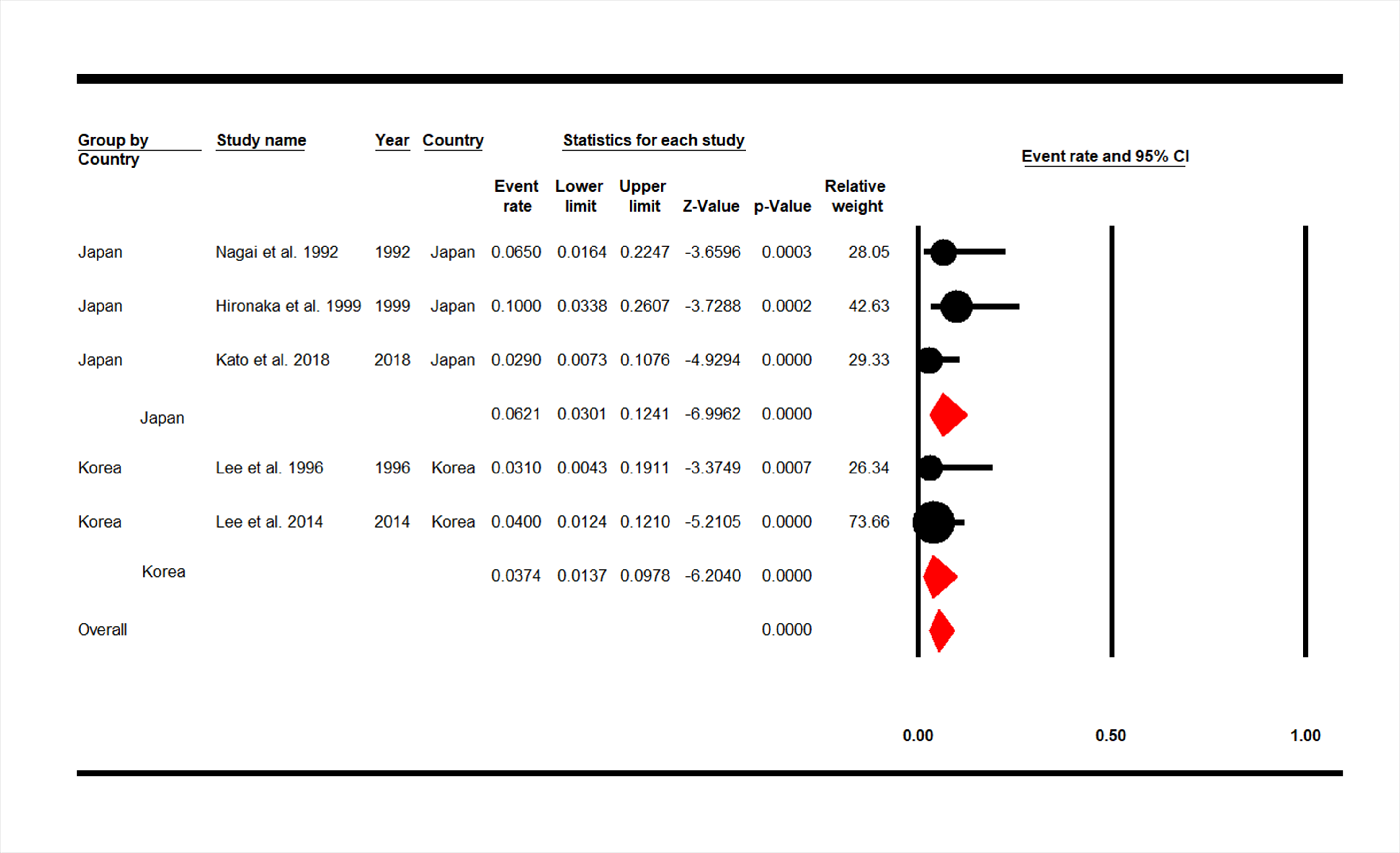

Supplement: S10 Fig — (TIF) [file pone.0202360.s015.tif]

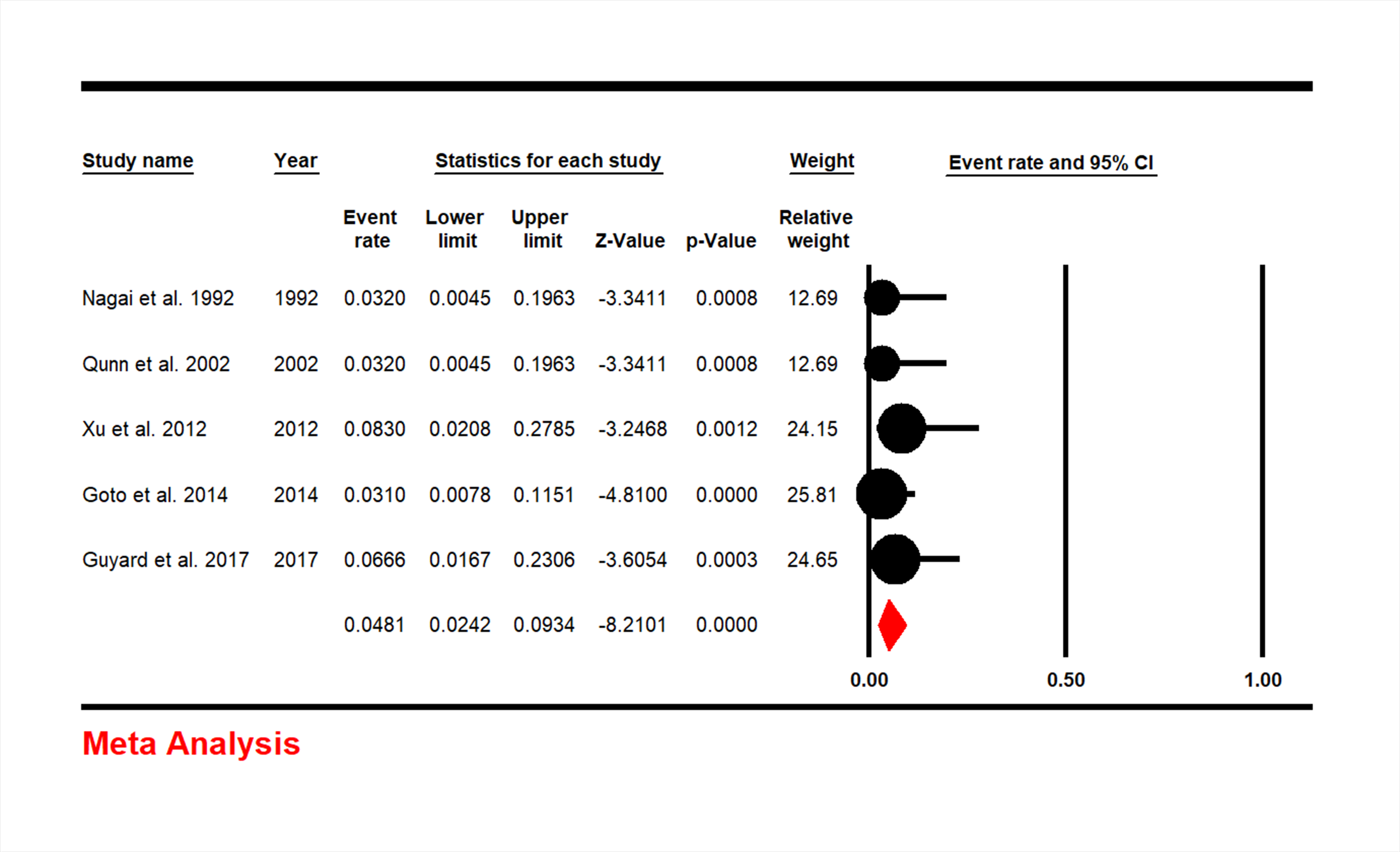

Supplement: S11 Fig — (TIF) [file pone.0202360.s016.tif]

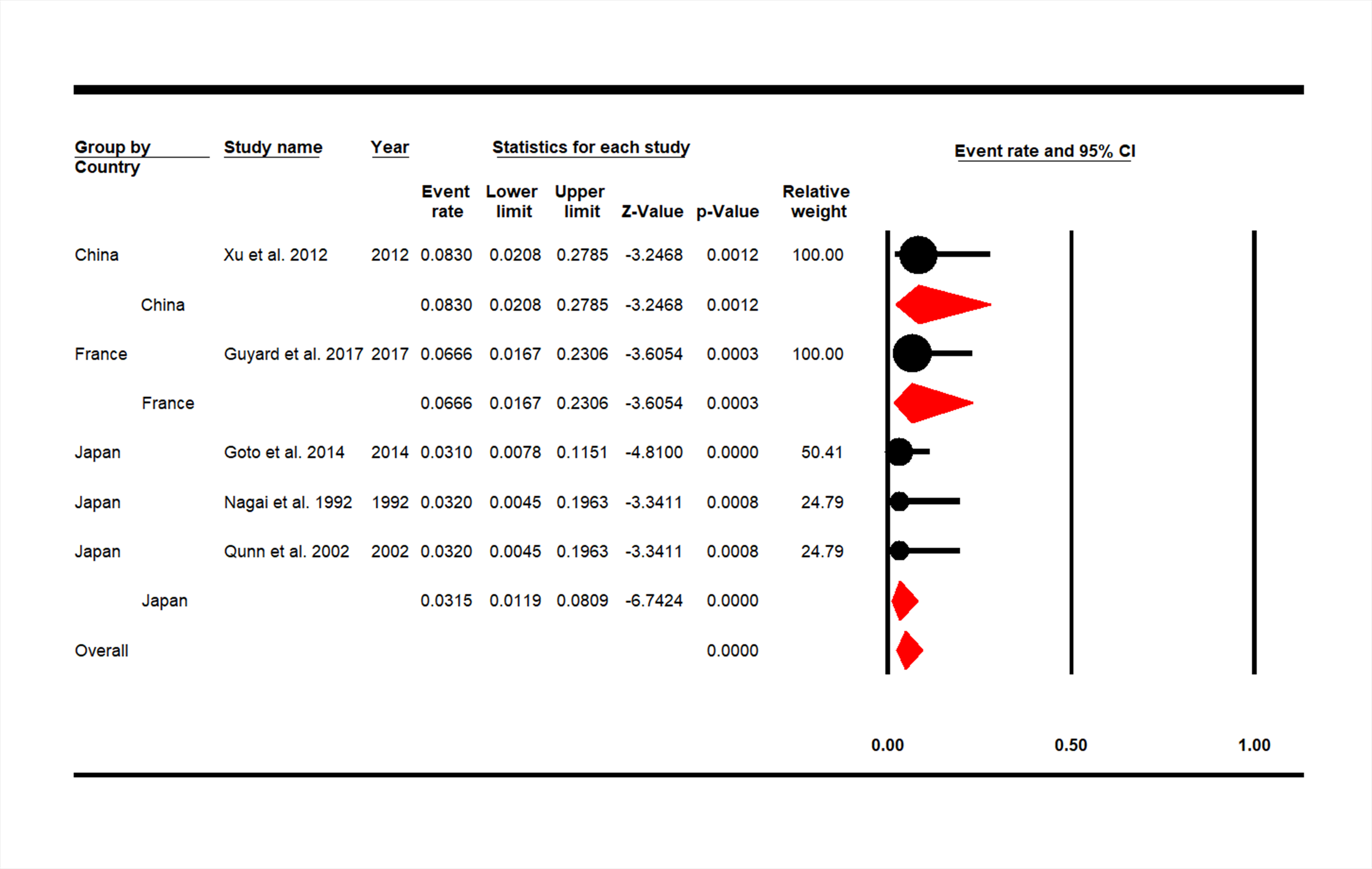

Supplement: S12 Fig — (TIF) [file pone.0202360.s017.tif]

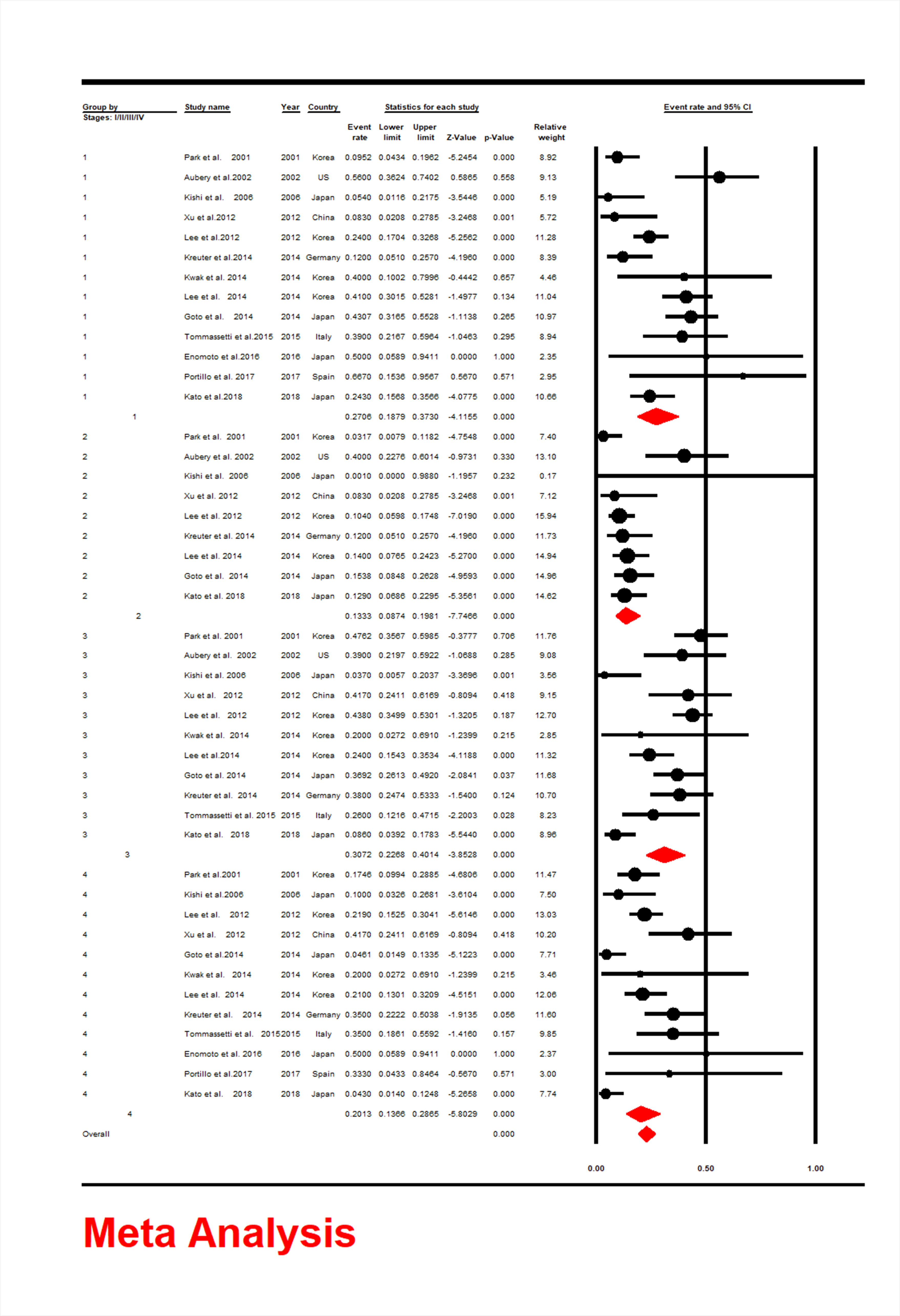

Supplement: S13 Fig — (TIF) [file pone.0202360.s018.tif]

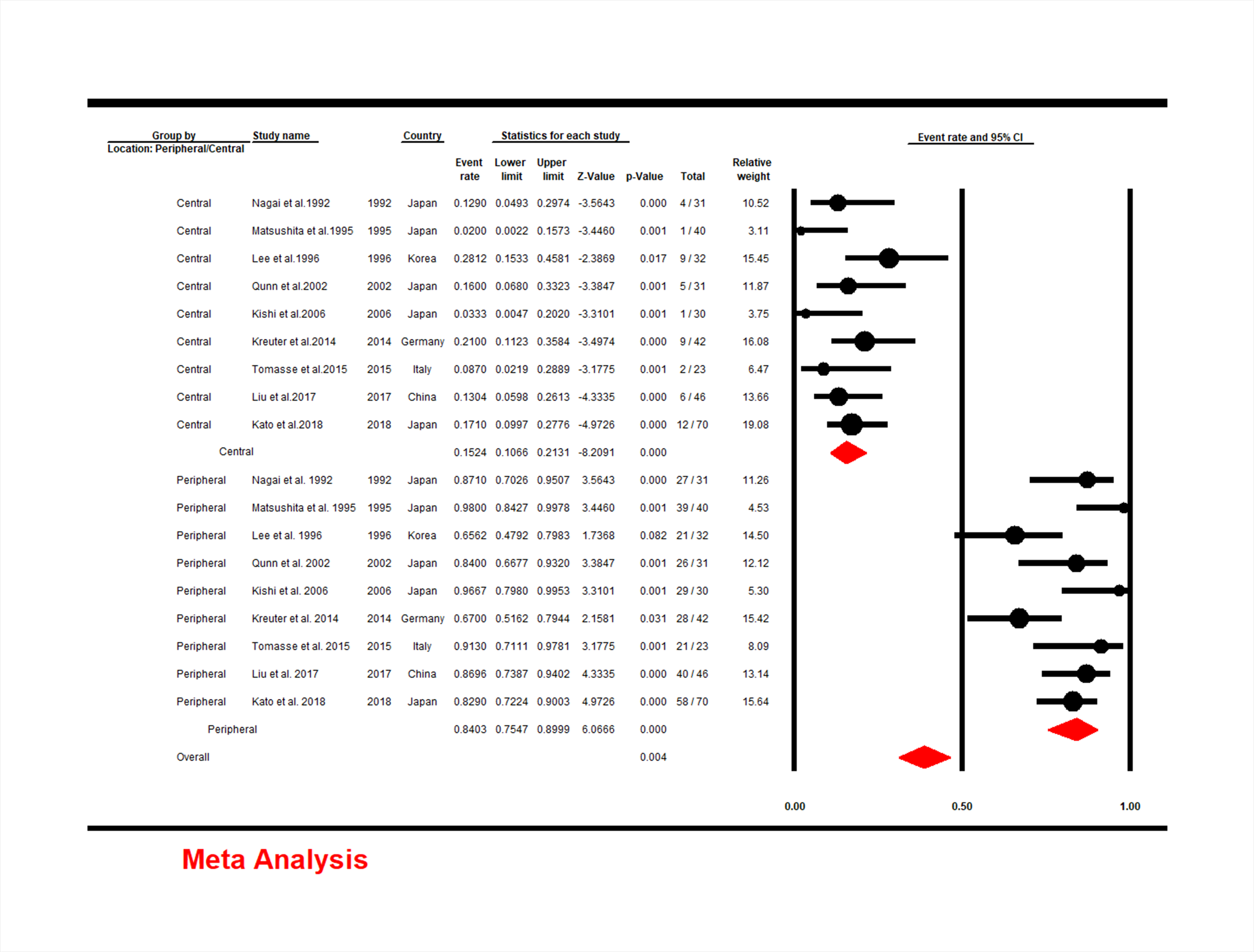

Supplement: S14 Fig — (TIF) [file pone.0202360.s019.tif]

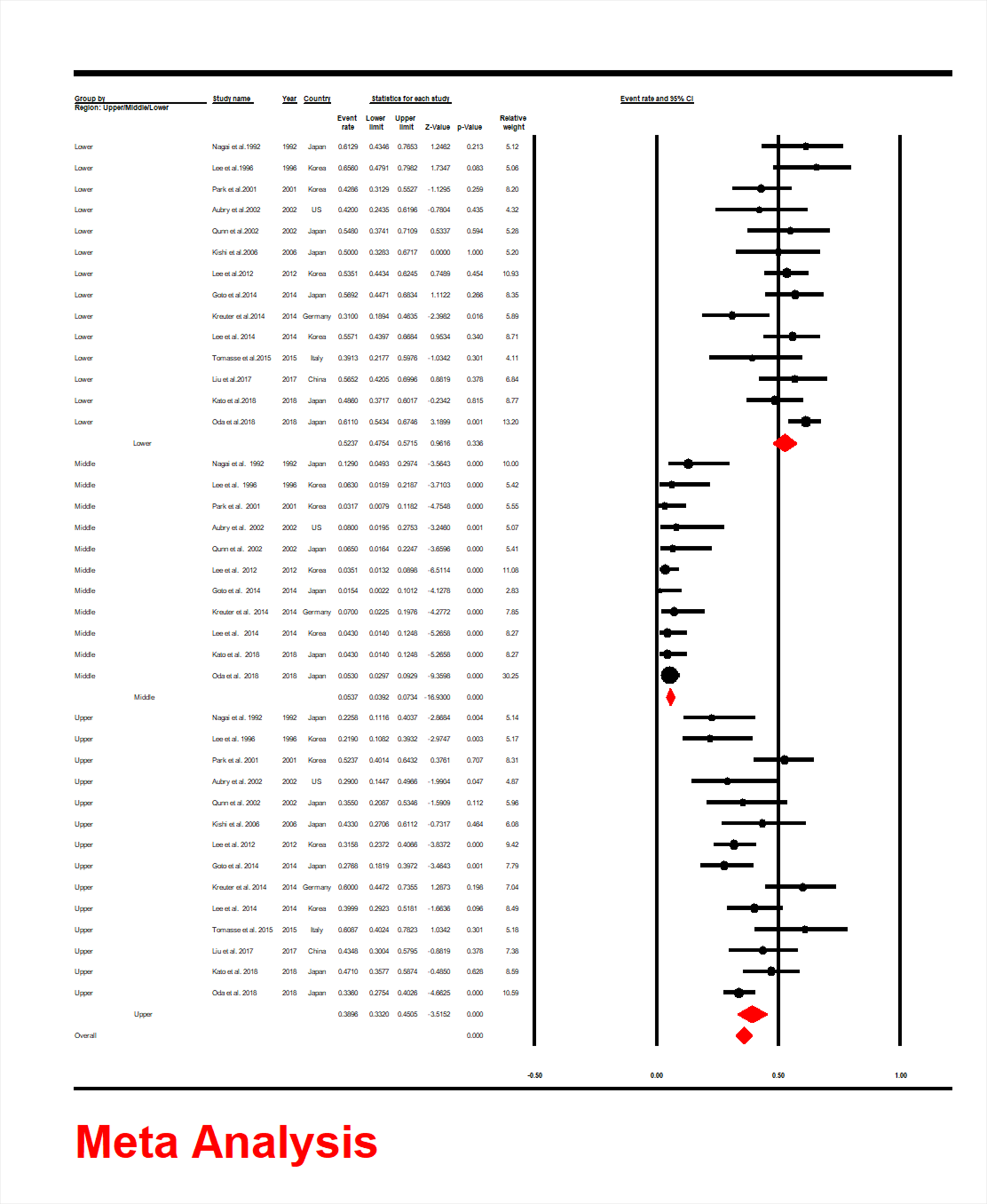

Supplement: S15 Fig — (TIF) [file pone.0202360.s020.tif]

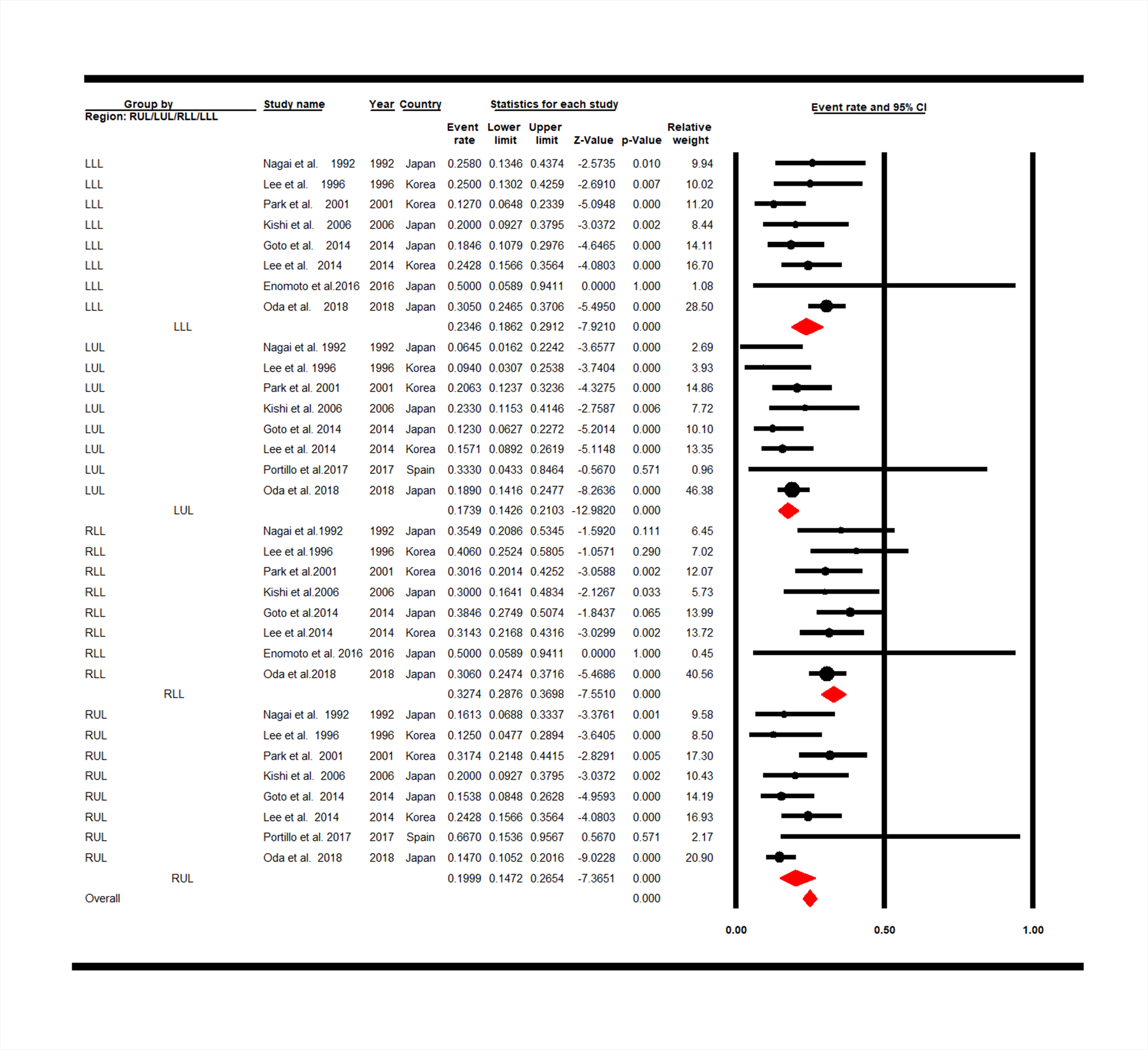

Supplement: S16 Fig — (TIF) [file pone.0202360.s021.tif]
